# The caseinolytic protease complex component CLPC1 in Arabidopsis maintains proteome and RNA homeostasis in chloroplasts

**DOI:** 10.1186/s12870-018-1396-0

**Published:** 2018-09-12

**Authors:** Shoudong Zhang, Huoming Zhang, Yiji Xia, Liming Xiong

**Affiliations:** 1 Department of Biology, Hong Kong Baptist University, Kowloon Tong, Hong Kong SAR, China; 2 Division of Biological and Environmental Sciences and Engineering, King Abdullah University of Science and Technology (KAUST), Thuwal, 23955-6900 Saudi Arabia; 3 Centre for Soybean Research, Partner State Key Laboratory of Agrobiotechnology and School of Life Sciences, The Chinese University of Hong Kong, Shatin, Hong Kong, Special Administrative Region China; 4 Core labs, King Abdullah University of Science and Technology (KAUST), Thuwal, 23955-6900 Saudi Arabia; 5 Partner State Key Laboratory of Environmental and Biological Analysis, Hong Kong Baptist University, Shatin, Hong Kong SAR, China; 6 Partner State Key Laboratory of Agrobiotechnology, Chinese University of Hong Kong, Shatin, Hong Kong, SAR, China; 7 Texas A&M AgriLife Research Center, Dallas, TX 75252 USA; 8 Department of Horticultural Sciences, Texas A&M University, College Station, TX 77843 USA

**Keywords:** Chloroplast, CLPC1, Proteome, Transcriptome, Homeostasis, SVR7

## Abstract

**Background:**

Homeostasis of the proteome is critical to the development of chloroplasts and also affects the expression of certain nuclear genes. CLPC1 facilitates the translocation of chloroplast pre-proteins and mediates protein degradation.

**Results:**

We found that proteins involved in photosynthesis are dramatically decreased in their abundance in the *clpc1* mutant, whereas many proteins involved in chloroplast transcription and translation were increased in the mutant. Expression of the full-length CLPC1 protein, but not of the N-terminus-deleted CLPC1 (ΔN), in the *clpc1* mutant background restored the normal levels of most of these proteins. Interestingly, the ΔN complementation line could also restore some proteins affected by the mutation to normal levels. We also found that that the *clpc1* mutation profoundly affects transcript levels of chloroplast genes. Sense transcripts of many chloroplast genes are up-regulated in the *clpc1* mutant. The level of SVR7, a PPR protein, was affected by the *clpc1* mutation. We showed that SVR7 might be a target of CLPC1 as CLPC1-SVR7 interaction was detected through co-immunoprecipitation.

**Conclusion:**

Our study indicates that in addition to its role in maintaining proteome homeostasis, CLPC1 and likely the CLP proteasome complex also play a role in transcriptome homeostasis through its functions in maintaining proteome homeostasis.

**Electronic supplementary material:**

The online version of this article (10.1186/s12870-018-1396-0) contains supplementary material, which is available to authorized users.

## Background

A chloroplast is an endosymbiotic organelle [1] that originated from a photoautotrophic bacterium. During evolution, most of endosymbiotic bacterial genes moved to the host genome [2], and only 5–10% of photoautotrophic bacterial genes stayed in the chloroplast genome [3]. As a consequence, the development and functions of chloroplasts depend heavily on host gene expression [4]. Proteins expressed from nucleus-encoded genes are synthesized as precursor proteins (pre-proteins) with amino terminal extension called transit peptides. The transit peptides will be proteolytically removed after their importing into chloroplast [5]. During the transport of these proteins into stroma of the chloroplast, the transient peptide forms a guide complex that includes the precursor protein (pre-protein), HSP70, and/or 14–3-3, and some unidentified proteins and docks at the outer envelope membrane of the chloroplast for translocation [6]. Translocation of pre-proteins across the envelope membrane is achieved by TOC (translocon at the outer envelope membrane of chloroplasts) and TIC (translocon at the inner envelope membrane of chloroplasts) complexes energized by ATP and GTP hydrolysis [6]. Nonetheless, not all plastid proteins are targeted via canonical, transit peptide-mediated engagement of the TOC–TIC machinery. Around 10% of chloroplast proteins have been estimated to arrive via non-canonical routes [7]. The CLPC1 (Clp protease ATP-binding subunit) protein (also known as HSP93v) was suggested to promote ATP hydrolysis to facilitate the functioning of the TIC complex [8]. Moreover, as a HSP100 molecular chaperone, it was suggested that CLPC1 participates in the CLP protease complex to degrade aggregated and mis-folded proteins [9, 10, 11]. Arabidopsis knockout mutants of CLPC1 were shown to have decreased efficiency of import and degradation of chloroplast proteins [12, 13]. These changes in protein homeostasis in chloroplasts may also affect gene expression in chloroplasts, although there has been limited study of this possibility.

Gene transcription and subsequent RNA processing in chloroplast are regulated both by chloroplast-encoded as well as by nucleus-encoded proteins [14, 15]. Besides Plastid-Encoded Polymerase (PEP) proteins such as rpoA, rpoB, rpoC1, and rpoC2 [16], gene transcription in chloroplasts requires Nucleus-Encoded RNA Polymerase (NEP) [17] especially when PEP activity is lacking. Moreover, the activity of PEP RNA polymerases also requires nucleus-encoded proteins such as pTACs (plastid transcriptionally active chromosome proteins) [18, 19] and sigma factors [20]. Unlike in eukaryotic genomes, the genes in the chloroplast genome are transcribed as polycistronic units and antisense RNAs can also be produced [21]. An important feature of chloroplast RNA metabolism is that large numbers of RNA-binding proteins are involved. In particular, hundreds of the so-called pentatricopeptide repeat proteins (PPR) are found to participate in RNA processing in chloroplasts. The functions of these proteins include binding RNAs to protect them from RNase J degradation and/or to facilitate or directly participate in their processing. Characterized PPR proteins include, for example, MRL1 (binding *rbcL* mRNA) [22], SVR7 (binding *ATPases* mRNAs) [23, 24], and HCF152 (binding *psbB-psbT-psbH-petB-petD* mRNAs) [14]. Other RNA-binding proteins are also involved in chloroplast RNA processing. For instance, CHLOROPLAST RNA-BINDING PROTEIN 29 (CP29), CP31 [25] and RNA helicase (RH3) [26] were suggested to function in group II intron splicing of chloroplast mRNAs. They also involved in rRNA processing, especially 23 s rRNA [26]. Various RNases (e.g., RNaseJ [27] and CSP41B [15]) mediate chloroplast RNA degradation and polycistronic RNA maturation. Due to the importance of these RNA-processing proteins, it seems likely that their dynamics may impact gene expression and function in chloroplasts.

A number of RNA metabolism proteins such as RH3, RNA-binding proteins, and some EF-Ts (translation elongation factors) were found to be over-accumulated in the *clpc1* mutant [11, 28]. In particular, Nishimura et al... (2013) analyzed proteomes of the *clpc1* mutant along with other *clp* mutants using a label free method, and proteins involved in chloroplast RNA metabolism and other functions and pathways were found to be differentially accumulated in these mutants [28]. These studies suggest that CLPC1 may be involved in the homeostasis of these proteins in chloroplasts. In this study, we used the iTRAQ (Isobaric tag for relative and absolute quantitation) method to analyze proteomes of not only the *clpc1* mutant and wild-type plants but also two different complementation lines (one expresses a truncated CLPC1 that lacks the 93 N-terminal amino acids (referred to as ΔN) and the other is a full-length CLPC1 complementary line (referred to as CP [29]). Our analysis led to the identification of additional proteins that displayed mis-regulation in the *clpc1* mutant. These include those involved in RNA metabolism, such as RNase J, several PEP components and PPR proteins. We also found that SVR7 (another PPR protein) was mis-regulated in the *clpc1* mutant. Our results indicate that CLPC1 also plays a direct or indirect role in chloroplast transcriptome homeostasis likely through its role in maintaining levels of proteins involved in transcription and RNA metabolism.

## Results

### iTRAQ based proteomics analysis identified new mis-regulated proteins in *clpc1* chloroplasts

**Fig. 1:**
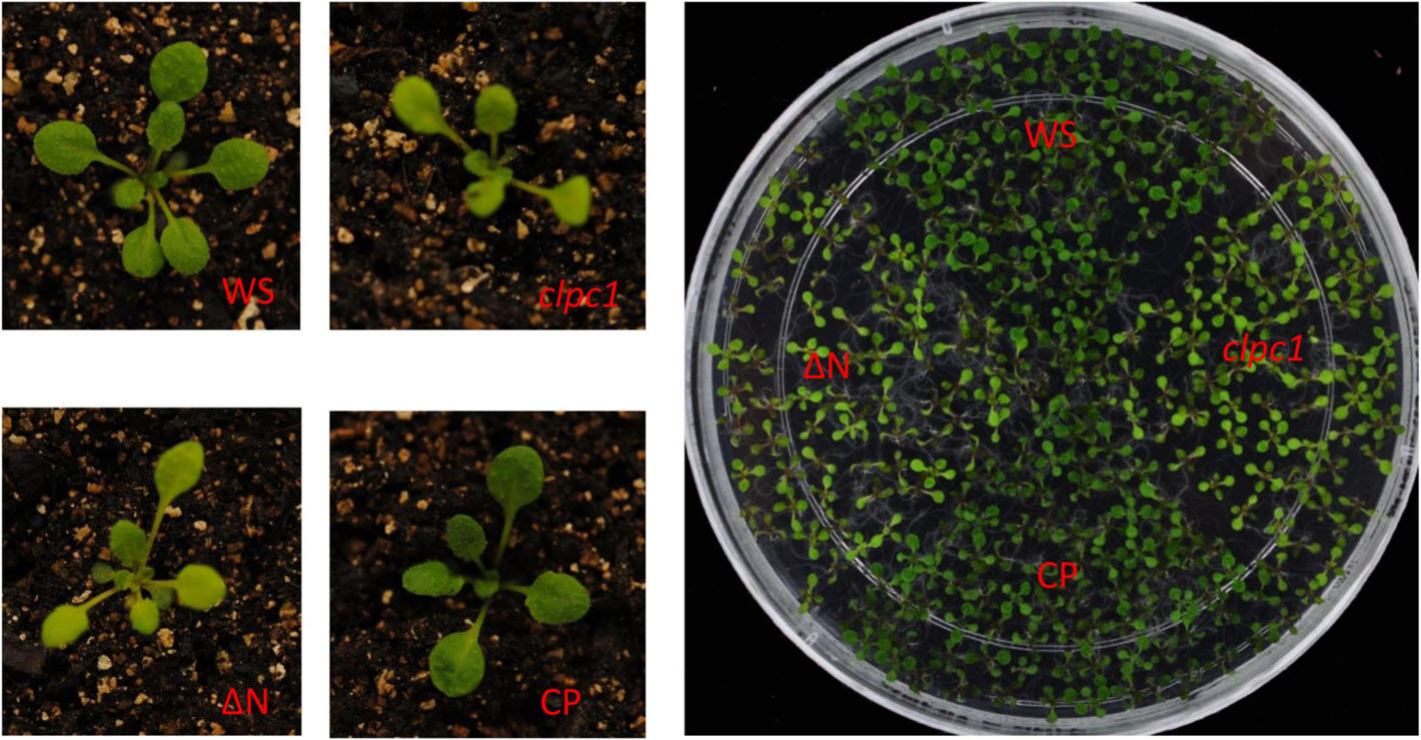
Morphology of the wild type, *clpc1*, ΔN as well as CP seedlings in soil (left panels) and in the medium (right panel). WS, the wild type (WS ecotype); *clpc1*, the *clpc1* mutant; ΔN, the *clpc* mutant expressing N-terminus-truncated CLPC1; CP, the *clpc* mutant expressing the full-length wild-type CLPC1

In Arabidopsis plastids (including chloroplasts), currently 2374 proteins have been identified according to the PPDB database [30]. Among them, the CLP protease complex is crucial to chloroplast development and embryogenesis [31]. CLP proteases are ATP-dependent caseinolytic proteases, consisting of a single proteolytic core complex with 11 distinct subunits including ClpP1, ClpP3–6, ClpR1–4, and ClpT1–2. Moreover, three potential chaperone partners ClpC1, ClpC2, and ClpD and an adaptor protein, CLPS [28] may facilitate the protease complex activity. A proteomics analysis indicated that some proteins in the *clpc1* mutant were mis-regulated. For example, photosystem proteins were found with reduced abundance whereas Hsp70, Cpn60, and some RNA-binding proteins were up-regulated [11]. The *clpc1* mutant in the WS background had similar morphological phenotypes to those of clpc1 in the Col-0 background such as pale green leaves and retarded growth33 (Fig. 1). Interestingly, the N-terminus-deleted CLPC1 (ΔN) could not complement these phenotypes but full-length CLPC1 could (Fig. 1)28.

To discover all CLPC1 functions in the proteome homeostasis in chloroplasts, we performed iTRAQ quantitative proteomic analyses on the chloroplasts from the clpc1 mutant (WS background)33, ΔN and the full-length CLPC1 complementation lines28 as well as the WS wild type (Fig. 1). As a result, we identified more than 800 proteins with almost all of them quantified (Additional file 1) from a total of 3 biological replicates. Among these, the first biological replicate samples were from 4-week-old, long-day, soil-grown seedlings, and its quantitative proteomics was based on three technical replicates. The other two biological replicates were from 2-week-old, long-day, soil-grown seedlings, and each biological replicate included 3 technical replicates. The mean and standard errors were based on the last two biological replicates (see Additional file 1: Figure S3). The Additional file 1 shows examples of spectra from the identified proteins. We considered proteins with a greater than 1.5-fold change as differentially expressed. These data not only confirmed the results of mis-regulated proteins in the *clpc1* mutant as previously reported [11] (Table 1a), but also demonstrated that the mis-regulated proteins resulted from the lack of a functional CLPC1 protein because in the full-length CLPC1 complementation line these proteins could be restored to the wild-type levels as the previous report [11, 28] did not include proteomic data of a complemented line. Moreover, our data indicated that the N-terminus deleted CLPC1 has partial functions in protein homeostasis since ΔN could restore or decrease the abundance of certain over-accumulated proteins in the *clpc1* mutant (Table 1a). It is interesting to note that a number of chloroplast RNA metabolism-related proteins were accumulated in the *clpc1* mutant (Tables 2 and 3).

**Table 1:** Chloroplast proteins over-accumulated in the *clpc1* mutant that were previously identified [11] in *clpc1* mutant (1a) or *clpp6* antisense line (1b) as putative targets of CLPP

		1st batch 4-week-old (LD)			2nd batch (2 biological replicates) 2-week-old (LD)			Nishimura et al (2013) 6-week-old (SD)
symbol	accession	*clpc1/WS*	*ΔN/WS*	*CP/WS*	*clpc1/WS*	*ΔN/WS*	*CP/WS*	*clpc1–1/wt*
1a. Previous identified misregulated proteins in clpcl mutant								
RH3	AT5G26742.1	8.3 ± 0.8*	3.0 ± 0.3	0.7 ± 0.1	2.6 ± 0.1*	2.0 ± 0.1	0.8 ± 0.1	1.0
CPN60A	AT2G28000.1	4.9 ± 0.2	3.6 ± 0.1	0.8 ± 0.1	1.9 ± 0.1	1.9 ± 0.1	0.7 ± 0.2	1.4
TCP-1	AT3G13470.1	4.7 ± 0.2	3.5 ± 0.1	0.8 ± 0.1	1.8 ± 0.0	2.0 ± 0.0	0.6 ± 0.1	1.7
HSP90.5	AT2G04030.1	4.0 ± 0.2	2.7 ± 0.2	0.9 ± 0.1	1.6 ± 0.1	1.5 ± 0.1	0.6 ± 0.0	1.6
cpHsc70–2	AT5G49910.1	3.7 ± 0.1*	2.8 ± 0.1	0.8 ± 0.1	1.8 ± 0.2*	1.8 ± 0.1	0.5 ± 0.0	1.1
cpHsc70–1	AT4G24280.1	3.7 ± 0.1*	2.9 ± 0.1	0.8 ± 0.1	1.8 ± 0.3*	1.9 ± 0.2	0.5 ± 0.0	1.0
RNA-binding	AT2G37220.1	3.5 ± 0.3	2.2 ± 0.2	0.8 ± 0.1	1.8 ± 0.0	1.9 ± 0.1	0.6 ± 0.1	2.3
EF-Ts	AT4G29060.1	3.5 ± 0.2*	2.2 ± 0.2	0.8 ± 0.1	1.4 ± 0.2	1.3 ± 0.1	0.6 ± 0.1	0.9
CPN21	AT5G20720.1	2.8 ± 0.2	3.1 ± 0.1	0.7 ± 0.1	1.8 ± 0.3	1.7 ± 0.2	0.5 ± 0.0	2.1
ATTIC110	AT1G06950.1	2.6 ± 0.2*	2.0 ± 0.1	0.9 ± 0.1	1.3 ± 0.0*	1.4 ± 0.0	0.7 ± 0.1	0.0
lipoxygenase 2	AT3G45140.1	1.5 ± 0.1*	1.0 ± 0.1	1.3 ± 0.1	1.5 ± 0.0*	1.6 ± 0.0	1.5 ± 0.2	1.0
AtcpSecA	AT4G01800.1	1.4 ± 0.1	1.4 ± 0.1	0.9 ± 0.1	1.0 ± 0.1	1.1 ± 0.1	0.6 ± 0.1	1.4
1b. Clpc1 mutant mis-regulated CLPP6 knocking down line identified targets								
RH3	AT5G26742.1	8.3 ± 0.8*	3.0 ± 0.3	0.7 ± 0.1	2.6 ± 0.1*	2.0 ± 0.1	0.8 ± 0.1	1.0
HSP90.5	AT2G04030.1	4.0 ± 0.2	2.7 ± 0.2	0.9 ± 0.1	1.6 ± 0.1	1.5 ± 0.1	0.6 ± 0.0	1.6
EF-Ts	AT4G29060.1	3.5 ± 0.2*	2.2 ± 0.2	0.8 ± 0.1	1.4 ± 0.2	1.3 ± 0.1	0.6 ± 0.1	0.9
FBA2	AT4G38970.1	2.9 ± 0.1*	2.0 ± 0.1	0.7 ± 0.1	1.4 ± 0.3	1.2 ± 0.1	0.5 ± 0.1	0.9
ATNDPK2	AT5G63310.1	2.8 ± 0.6	2.3 ± 0.5	0.8 ± 0.1	1.3 ± 0.0	1.3 ± 0.0	0.6 ± 0.1	1.2
Ribose 5-phospha	AT3G04790.1	2.7 ± 0.3	2.0 ± 0.3	0.8 ± 0.1	1.1 ± 0.0	1.1 ± 0.1	0.6 ± 0.2	1.0
ROC4	AT3G62030.1	1.8 ± 0.1	1.5 ± 0.1	0.9 ± 0.1	1.1 ± 0.1	1.3 ± 0.1	0.3 ± 0.0	0.9

WS: wild type; *clpc1*: the *clpc1* mutant; ΔN, N-terminal (1–93 amino acid) deleted CLPC1 complementary line; CP, full-length CLPC1 complementary line, LD, long-day; SD, short-day

The first batch dataset was from 4-week-old seedlings with three technical replicates. The second batch was from 2-week-old seedlings with two biological replicates. Each biological replicate included 3 technical replicates

Data are means and standard errors of protein abundance relative to the wild type (WS)

Data from Nishimura et al... (2013) in [28]

Proteins marked with * are significantly (*p* < 0.05) over- or under-accumulated in the *clpc1* mutant in the current study that were not significantly altered or not detected in a previous study (Nishimura et al..... 2013)

**Table 2:** Chloroplast RNA metabolism proteins in the *clpc1* mutant and the complementary lines

		1st batch 4-week-old (LD)			2nd batch (2 biological replicates) 2-week-old (LD)			Nishimura et al (2013) 6-week-old (SD)
		*clpc1/WS*	*ΔN/WS*	*CP/WS*	*clpc1/WS*	*ΔN/WS*	*CP/WS*	*clpc1–1/wt*
PPR proteins								
MEE40	AT3G53700.1	3.1 ± 1.4*	2.6 ± 1.0	1.1 ± 0.1	2.4 ± 0.3*	2.2 ± 0.0	1.1 ± 0.0	n.a
SVR7	AT4G16390.1	3.0 ± 0.3*	2.5 ± 0.3	1.1 ± 0.2	1.7 ± 0.0*	1.7 ± 0.0	0.7 ± 0.0	1.0
MRL1	AT4G34830.1	3.9 ± 0.4*	3.1 ± 0.7	1.0 ± 0.1	1.4 ± 0.5	1.6 ± 0.6	1.1 ± 0.1	n.a
PEP proteins								
RPOA	ATCG00740.1	3.0 ± 0.4*	3.0 ± 0.2	0.9 ± 0.1	2.0 ± 0.1*	1.9 ± 0.1	1.5 ± 0.1	n.a
RPOB	ATCG00190.1	3.3 ± 0.6*	3.9 ± 0.9	1.3 ± 0.1	1.5 ± 0.0*	1.7 ± 0.0	1.6 ± 0.2	n.a
RPOC2	ATCG00170.1	n.a	n.a	n.a	1.5 ± 0.1*	1.6 ± 0.0	1.5 ± 0.3	n.a
RNA binding proteins								
RH3	AT5G26742.1	8.3 ± 0.8*	3.0 ± 0.3	0.7 ± 0.1	2.6 ± 0.1*	2.0 ± 0.1	0.8 ± 0.1	1.0
RNA-binding	AT1G70200.1	4.9 ± 1.7*	3.4 ± 0.8	0.9 ± 0.1	3.0 ± 0.2*	2.9 ± 0.1	1.1 ± 0.0	n.a
RNA-binding	AT4G09040.2	4.8 ± 0.6	3.8 ± 0.5	1.1 ± 0.1	1.8 ± 0.2	1.6 ± 0.1	0.6 ± 0.0	596.1
RNA-binding	AT3G52150.1	4.4 ± 0.6*	2.7 ± 0.5	0.8 ± 0.1	2.3 ± 0.5*	2.1 ± 0.4	0.5 ± 0.1	n.a
CP29	AT3G53460.1	4.1 ± 0.5	2.3 ± 0.2	1.0 ± 0.1	2.0 ± 0.4	1.7 ± 0.2	0.5 ± 0.0	5.4
RNA-binding	AT1G73530.1	4.1 ± 0.1*	3.3 ± 0.1	0.9 ± 0.1	1.6 ± 0.3*	1.3 ± 0.2	1.2 ± 0.0	n.a
RNA-binding	AT2G35410.1	3.1 ± 0.3	2.7 ± 0.2	0.9 ± 0.1	1.4 ± 0.2	1.4 ± 0.1	0.6 ± 0.1	1.6
CP33	AT3G52380.1	2.6 ± 0.1	1.9 ± 0.1	0.8 ± 0.1	1.2 ± 0.0	1.2 ± 0.0	0.6 ± 0.1	0.9
RBP31	AT4G24770.1	2.4 ± 0.1	1.6 ± 0.1	0.8 ± 0.1	1.0 ± 0.0	1.2 ± 0.0	0.8 ± 0.1	0.8
RNA modification protein								
16S rRNA process	AT5G46420.1	4.7 ± 1.6*	3.8 ± 13	0.9 ± 0.6	2.3 ± 0.1*	2.0 ± 0.1	0.8 ± 0.0	n.a
RIF10, PNPase	AT3G03710.1	4.1 ± 0.5	3.1 ± 0.5	0.9 ± 0.1	1.5 ± 0.1	1.6 ± 0.0	0.8 ± 0.0	1.6
RNA 3′ phosphate	AT1G48860.1	2.6 ± 0.0*	1.5 ± 0.0	1.1 ± 0.0	1.5 ± 0.2*	1.0 ± 0.2	0.5 ± 0.1	0.9
RNAses								
RNAse J	AT5G63420.1	3.0 ± 0.2*	2.8 ± 0.1	1.1 ± 0.2	1.4 ± 0.0*	1.7 ± 0.1	0.9 ± 0.2	n.a
CSP41B	AT1G09340.1	2.3 ± 0.1	1.3 ± 0.1	0.7 ± 0.1	1.0 ± 0.1	0.8 ± 0.1	0.5 ± 0.1	0.4
PRORP1	AT2G32230.1	1.9 ± 0.0*	1.8 ± 0.0	0.9 ± 0.0	1.7 ± 0.0*	1.8 ± 0.1	1.0 ± 0.3	n.a

Notes: See notes of Table 1

**Table 3:** Relative abundance of the pTAC proteins in *clpc1* and its complementary lines

		1st batch 4-week-old (LD)			2nd batch (2 biological replicates) 2-week-old (LD)			Nishimura et al (2013) 6-week-old (SD)
symbol	accession	*clpcl/WS*	*ΔN/WS*	*CP/WS*	*clpc1/WS*	*ΔN/WS*	*CP/WS*	*clpc1–1/wt*
PTAC14	AT4G20130.1	3.7 ± 0.5*	2.6 ± 0.2	1.2 ± 0.1	2.1 ± 0.0*	1.8 ± 0.1	1.5 ± 0.3	n.a
PTAC13	AT3G09210.1	3.7 ± 0.0*	3.8 ± 0.0	0.8 ± 0.0	1.7 ± 0.1*	1.8 ± 0.0	1.0 ± 0.1	n.a
PTAC11	AT2G02740.1	3.3 ± 0.1*	3.4 ± 0.3	0.9 ± 0.1	1.7 ± 0.1*	1.9 ± 0.0	1.1 ± 0.1	n.a
PTAC2	AT1 G74850.1	2.7 ± 0.3*	1.8 ± 0.1	1.1 ± 0.2	1.7 ± 0.2*	1.6 ± 0.2	1.4 ± 0.1	n.a
PTAC10	AT3G48500.1	2.5 ± 0.3*	2.4 ± 0.3	1.0 ± 0.1	1.8 ± 0.2*	1.7 ± 0.1	1.4 ± 0.0	n.a
PTAC4	AT1 G65260.1	2.2 ± 0.2*	2.0 ± 0.2	1.0 ± 0.1	1.4 ± 0.1	1.3 ± 0.1	1.0 ± 0.1	n.a
PTAC17	AT1 G80480.1	2.0 ± 0.2	1.5 ± 0.1	0.9 ± 0.1	1.4 ± 0.1	1.3 ± 0.1	0.77 ± 0.0	2.4
PTAC5	AT4G13670.1	1.8 ± 0.1*	2.0 ± 0.1	1.0 ± 0.1	1.6 ± 0.0*	1.7 ± 0.1	1.2 ± 0.2	n.a

Notes: See notes of Table 1

CLPP6 is one core component of the heptameric P-ring of the CLPRT protease complex. It has been shown that the CLPP6 antisense line had a distinct protein expression profile compared with the wild type, and thus defined the CLP protease complex targets [32]. A putative function of CLPC1 is to facilitate the CLPRT protease complex to degrade its targets via the CLPC1 chaperone activity [11]. Therefore, we predict that the CLPP6 antisense line might share some common mis-regulated targets with the *clpc1* mutant. Indeed, we found that most of the previously reported over-accumulated proteins in the CLPP6 antisense line [11] also exhibited higher abundance in the *clpc1* mutant (Table 1b). However, other subunits of the CLPP complex, such as CLPP3, CLPP5, CLPR1, and CLPR3 that had less abundance in the CLPP6 antisense line [32] and the *clpr2* knockdown line [33], actually accumulated more in the *clpc1* mutant relative to the wild type, similar to what was reported [28] (Table 4a). These components of the CLPP complex were also accumulated more in the *clpp3* knock-out line where the level of both CLPC1 and CLPC2 proteins was reduced [31]. These over-accumulated subunits of the CLP protease complex include all the core components of the complex (Table 4a). Notably, the differentially accumulated proteins in the *clpc1* mutant can be restored to the wild-type level in the full-length CLPC1 complementation lines.

**Table 4:** Relative abundance of protein components in the CLPP and translocon complexes

		1st batch 4-week-old (LD)			2nd batch (2 biological replicates) 2-week-old (LD)			Nishimura et al (2013) 6-week-old (SD)
symbol	accession	*clpcl/WS*	*ΔN/WS*	*CP/WS*	*clpcl/WS*	*ΔN/WS*	*CP/WS*	*clpc1–1/wt*
a. CLPP components								
CLPP1	ATCG00670.1	4.7 ± 0.3	3.2 ± 0.1	1.0 ± 0.2	1.5 ± 0.0	1.2 ± 0.2	1.0 ± 0.0	1.2
ClpR3	AT1G09130.1	4.6 ± 1.1	2.5 ± 0.6	0.7 ± 0.1	1.8 ± 0.0	1.4 ± 0.1	0.7 ± 0.2	1.4
ClpT1	AT4G25370.1	4.4 ± 0.3	2.8 ± 0.2	0.9 ± 0.1	1.9 ± 0.7	1.2 ± 0.4	1.0 ± 0.0	2.1
CLPR1	AT1G49970.1	4.1 ± 1.5	2.3 ± 0.5	0.9 ± 0.1	2.3 ± 0.9	1.5 ± 0.4	0.7 ± 0.0	1.9
CLPC2	AT3G48870.2	4.0 ± 1.1	3.1 ± 0.5	1.4 ± 0.1	1.5 ± 0.1	1.7 ± 0.0	0.8 ± 0.0	2.5
CLPR2	AT1G12410.1	3.8 ± 0.3	2.7 ± 0.1	0.8 ± 0.1	2.4 ± 0.7	1.5 ± 0.4	0.8 ± 0.1	2.1
CLPP4	AT5G45390.1	3.8 ± 0.2	2.6 ± 0.2	0.7 ± 0.1	2.0 ± 0.3	1.6 ± 0.3	0.7 ± 0.1	1.3
CLPP6	AT1G11750.1	3.4 ± 0.3	2.9 ± 0.3	0.9 ± 0.1	1.7 ± 0.5	1.7 ± 0.3	0.7 ± 0.1	3.6
CLPP3	AT1G66670.1	3.3 ± 0.3*	2.6 ± 0.2	0.9 ± 0.2	1.5 ± 0.2*	1.3 ± 0.1	0.7 ± 0.3	1.0
CLPR4	AT4G17040.1	3.1 ± 1.0	2.0 ± 0.4	0.9 ± 0.1	2.0 ± 0.2	1.6 ± 0.1	0.7 ± 0.2	1.5
ClpT2	AT4G12060.1	3.0 ± 0.2	2.1 ± 0.1	0.9 ± 0.1	1.9 ± 0.2	1.5 ± 0.1	1.1 ± 0.1	1.4
CLPB3	AT5G15450.1	2.3 ± 0.9	1.6 ± 0.2	1.1 ± 0.1	2.3 ± 0.3	1.5 ± 0.1	0.7 ± 0.0	3.4
b. TIC/TOC complex components								
TIC40	AT5G16620.1	3.1 ± 0.3*	2.4 ± 0.3	0.9 ± 0.1	1.6 ± 0.0*	1.9 ± 0.0	0.9 ± 0.0	n.a
ATTIC 110	AT1G06950.1	2.6 ± 0.1*	2.0 ± 0.1	0.9 ± 0.1	1.3 ± 0.0*	1.4 ± 0.0	0.7 ± 0.1	0.0
TOC64-III	AT3G17970.1	2.2 ± 0.0*	2.0 ± 0.0	1.1 ± 0.0	1.3 ± 0.1	1.2 ± 0.1	0.9 ± 0.0	n.a
TOC33	AT1G02280.1	2.2 ± 0.1*	2.2 ± 0.1	0.8 ± 0.1	1.1 ± 0.1	1.5 ± 0.0	0.9 ± 0.1	n.a
TOC 159	AT4G02510.1	1.8 ± 0.3	1.9 ± 0.2	1.0 ± 0.2	1.1 ± 0.0	1.5 ± 0.0	0.9 ± 0.0	1.1
TIC55-IV	AT4G25650.1	1.5 ± 0.1*	1.7 ± 0.1	1.5 ± 0.1	1.6 ± 0.1*	1.6 ± 0.0	1.3 ± 0.1	n.a
TIC55-II	AT2G24820.1	1.5 ± 0.1*	1.7 ± 0.1	1.5 ± 0.1	1.6 ± 0.1*	1.6 ± 0.0	1.3 ± 0.1	n.a
TOC75-III	AT3G46740.1	1.3 ± 0.1	1.6 ± 0.1	1.2 ± 0.1	1.2 ± 0.1	1.4 ± 0.1	0.8 ± 0.0	n.a

Notes: See notes of Table 1

Besides participating in the degradation of chloroplast proteins, CLPC1 was suggested to be involved in importing pre-proteins with inner membrane translocation complex components such as TIC110 and TIC40 [29, 34]. Our data showed that both TIC40 and TIC110 were over-accumulated in the *clpc1* mutant and in the ΔN line, and their levels were restored to those of the wild type in the full-length CLPC1 complementation line (Table 4b). In accordance to the import function of TIC110 and TIC40, the *clpc1* mutant also accumulated more stromal proteins Hsc70–1 and Hsc70–2, both of which are known to mediate pre-protein transport and folding following pre-protein TIC complex transport [34, 35] (Table 1a).

### Accumulation of chloroplast RNA metabolism proteins in the *clpc1* mutant

RNA homeostasis in chloroplasts is sustained by its biogenesis and degradation and mediated by chloroplast RNA polymerases, RNA-binding proteins, RNases and other proteins. We found that most of these RNA metabolism-related proteins were over-accumulated in the *clpc1* mutant as well as in the ΔN plants (Table 2). These proteins include PPR proteins (MEE40, SVR7, and MRL1), RNA-binding proteins (CP29, CP33, RH3, etc.), chloroplast RNases (PRORP1, RNAse J, CSP41B), as well as RNA modification proteins (RNA 3′-end phosphate cyclase, RIF10, and 16S rRNA processing protein). In the full-length CLPC1 complementation line, most of these proteins were restored almost to the wild-type level (Table 2). These results suggest that CLPC1 may have functions in maintaining the homeostasis of these RNA metabolism factors, likely by degrading them when they are damaged or over-accumulated.

Besides the above nucleus-encoded, chloroplast-localized RNA metabolism proteins, all plastid-encoded RNA polymerase (PEP) subunits identified in our proteomic profiling are also over-accumulated in the *clpc1* mutant. In addition, several plastid transcriptionally active chromosome proteins (pTACs), which facilitate PEP transcription [18], accumulated in the *clpc1* mutant, and their levels could be restored to those of the wild type by reintroducing the full-length CLPC1 into the mutant (Table 3). However, for unknown reasons, rpoA, rpoB, and rpoC2 did not restore to the wild type level in the 2-week-old samples and remained at a relative high level in the full-length CLPC1 complementary line (CP line) (Table 2).

### Accumulation of transcripts of chloroplast genes in the *clpc1* mutant

**Fig. 2:**
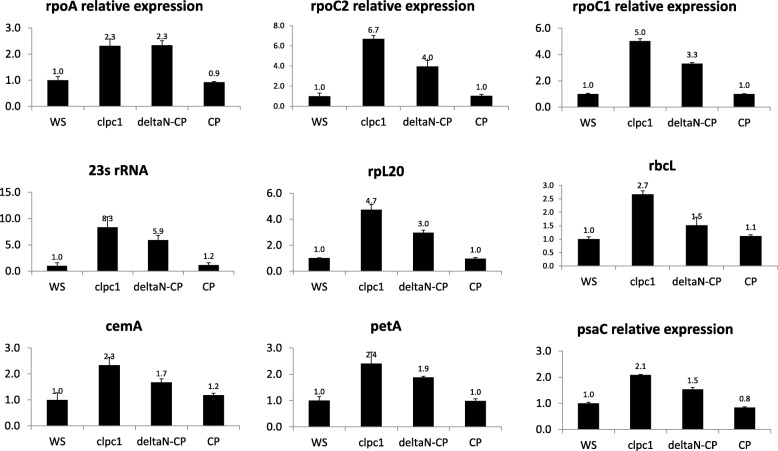
Relative expression levels of sense transcripts in the *clpc1* mutant and its complementation lines. Shown are means and SDs from three replicates. qRT-PCR was conducted using gene-specific primers (Additional file 1: Table S2) normalized against the expression of the *ACTIN2* Gene. WS, the wild type; *clpc1*, the *clpc1* mutant; ΔN, *clpc1* expressing N-terminus-truncated CLPC1; CP, *clpc1* expressing the full-length wild-type CLPC1

Transcription of the plastid genome is accomplished by two different phage-type RNA polymerases (NEP) (RPOTp and RPOTmp) [36–38] along with one eubacterial-type RNA polymerase (PEP) consisting of rpoA, rpoB, rpoC1 and rpoC2 subunits [39, 40]. The activity of PEP is regulated by six sigma-type nucleus-encoded transcription initiation factors [16, 41–44]. Nonetheless, the level of chloroplast transcripts is determined both by transcription and by their metabolism regulated by many RNA processing factors [22]. In our proteomics profiling, we found that the PEP proteins were over-accumulated in the *clpc1* mutant. Several PPR proteins, RNA-binding proteins, and RNA modification and degradation proteins were also over-accumulated in the mutant (Table 2). Similarly, there were several over-accumulated pTACs (Table 3). These data imply that CLPC1 may play a role in chloroplast RNA homeostasis. To test this hypothesis, we used gene-specific primers to perform qRT-PCR to specifically examine the level of sense transcripts in the wild type, *clpc1* mutant, and the two complementation lines. Our results showed that all chloroplast sense transcripts examined were over-accumulated in the *clpc1* mutant and the ΔN line, while they remained at the wild-type levels in the full-length CLPC1 complementation line (Fig. 2, Additional file 1: Figure S1).

### Decoupling of the transcript levels and protein levels in the chloroplast photosystem genes

The level of steady-state transcripts has often been used as a meter to indicate the level of gene expression when the protein level cannot be conveniently assessed. Indeed, in the current study, the over-accumulation of many sense transcripts of chloroplast genes correlated with an increased level of the corresponding proteins (Table 5a). However, this correlation does not always hold. In the *clpc1* mutant, certain genes with increased transcript levels were actually accompanied by dramatically reduced protein levels. These include most chloroplast-encoded photosystem proteins (see Table 5b) and ATPases. The reduced protein levels regardless of the high transcript levels (Fig. 2, Additional file 1: Figure S1) might have been caused by increased protease activities or by post-transcriptional regulation [45]. We did find that the levels of most of the CLPP subunits and other proteases, such as DEGp2, FTSH12, LON, were dramatically increased (Table 4a), although the levels of some other proteases (DEGP1, RD21, ARASP) decreased in the *clpc1* mutant (Additional file 1: Table S1). The higher level of proteases in the mutant might thus contribute to the down regulation of these photosystem proteins. It has been shown that the degradation of photosystem proteins is not dependent on energy [46] and therefore may not need CLPC1.

**Table 5:** Over- and under-accumulated chloroplast-encoded proteins in the *clpc1* mutant and its complementation lines

		1st batch 4-week-old (LD)			2nd batch (2 biological replicates) 2-week-old (LD)			Nishimura et al (2013) 6-week-old (SD)
SYMBOL	ID	*clpc1/WS*	*ΔN/WS*	*CP/WS*	*clpci/ws*	*ΔN/WS*	*CP/WS*	*clpc1–1/wt*
a. over-accumulated								
RPS4	ATCG00380.1	6.5 ± 0.4	4.9 ± 0.3	0.7 ± 0.1	1.8 ± 0.2	1.9 ± 0.0	0.8 ± 0.1	18.6
CLPP1	ATCG00670.1	4.7 ± 0.3	3.2 ± 0.1	1.0 ± 0.2	1.5 ± 0.0	1.2 ± 0.2	0.9 ± 0.2	1.2
RPS15	ATCG01120.1	3.4 ± 0.3*	2.8 ± 0.2	0.8 ± 0.1	1.7 ± 0.5	1.4 ± 0.1	1.1 ± 0.4	1.0
RPL22	ATCG00810.1	3.4 ± 0.3*	2.9 ± 0.3	0.9 ± 0.1	2.1 ± 0.1*	1.9 ± 0.1	1.0 ± 0.0	n.a
RPS8	ATCG00770.1	3.3 ± 0.4	2.5 ± 0.4	0.8 ± 0.1	1.8 ± 0.5	1.5 ± 0.2	1.0 ± 0.1	10.7
RPOB	ATCG00190.1	3.3 ± 0.6*	3.9 ± 0.9	1.3 ± 0.1	1.5 ± 0.0*	1.7 ± 0.0	1.6 ± 0.2	n.a
RPS18	ATCG00650.1	3.2 ± 0.4*	2.6 ± 0.1	0.8 ± 0.1	1.6 ± 0.1*	1.7 ± 0.1	1.0 ± 0.2	n.a
RPS2	ATCG00160.1	3.2 ± 0.1	2.8 ± 0.1	0.9 ± 0.1	1.6 ± 0.6	1.5 ± 0.4	0.9 ± 0.2	4.5
RPS7	ATCG00900.1	3.1 ± 0.2	2.6 ± 0.2	0.8 ± 0.1	1.6 ± 0.2	1.5 ± 0.1	1.0 ± 0.3	175.6
RPS3	ATCG00800.1	3.0 ± 0.2*	2.5 ± 0.2	1.0 ± 0.1	1.8 ± 0.0*	1.7 ± 0.0	0.8 ± 0.1	0.9
RPOA	ATCG00740.1	3.0 ± 0.4*	3.0 ± 0.2	0.9 ± 0.1	2.0 ± 0.1*	1.9 ± 0.1	1.5 ± 0.1	n.a
RPS14	ATCG00330.1	2.9 ± 0.6	2.3 ± 0.4	0.9 ± 0.1	1.0 ± 0.7	1.4 ± 0.2	1.2 ± 0.4	n.a
RPL20	ATCG00660.1	2.7 ± 0.5*	2.8 ± 0.6	0.8 ± 0.1	1.9 ± 1.1*	1.5 ± 0.1	3.6 ± 2.3	n.a
RPL14	ATCG00780.1	2.6 ± 0.2	2.7 ± 0.3	0.9 ± 0.1	1.9 ± 0.2	1.7 ± 0.2	0.5 ± 0.0	1.6
RPS19	ATCG00820.1	2.6 ± 0.5*	2.3 ± 0.4	0.8 ± 0.1	2.5 ± 0.8*	1.5 ± 0.1	1.2 ± 0.1	n.a
ACCD	ATCG00500.1	2.5 ± 0.2*	2.2 ± 0.2	1.0 ± 0.1	1.5 ± 0.1	1.3 ± 0.1	0.8 ± 0.0	n.a
RPL16	ATCG00790.1	2.2 ± 0.2*	2.1 ± 0.1	1.0 ± 0.1	1.3 ± 0.2	1.2 ± 0.0	1.7 ± 0.1	n.a
RPOC2	ATCG00170.1	n.a	n.a	n.a	1.5 ± 0.1	1.6 ± 0.0	1.5 ± 0.3	n.a
b. under-accumulated								
PSAB	ATCG00340.1	0.4 ± 0.1*	0.5 ± 0.1	1.1 ± 0.1	0.6 ± 0.1*	0.7 ± 0.1	2.6 ± 0.6	n.a
PSBC	ATCG00280.1	0.4 ± 0.1*	0.5 ± 0.1	1.0 ± 0.1	0.6 ± 0.1*	0.7 ± 0.0	2.9 ± 0.1	n.a
PSAA	ATCG00350.1	0.4 ± 0.1*	0.5 ± 0.1	1.2 ± 0.1	0.6 ± 0.2	0.8 ± 0.3	2.6 ± 1.0	n.a
PETB	ATCG00720.1	0.4 ± 0.1	0.5 ± 0.1	1.2 ± 0.1	0.4 ± 0.0	0.5 ± 0.0	1.2 ± 0.1	1.0
PSBA	ATCG00020.1	0.3 ± 0.1*	0.6 ± 0.1	1.0 ± 0.1	0.7 ± 0.1	0.7 ± 0.1	2.3 ± 0.2	n.a
PSBD	ATCG00270.1	0.3 ± 0.1*	0.5 ± 0.1	1.0 ± 0.1	0.8 ± 0.1	0.8 ± 0.0	2.8 ± 0.1	n.a
PSBB	ATCG00680.1	0.3 ± 0.1*	0.5 ± 0.1	1.1 ± 0.1	0.6 ± 0.1*	0.7 ± 0.1	2.2 ± 0.5	n.a
PSBH	ATCG00710.1	0.3 ± 0.1*	0.5 ± 0.1	0.8 ± 0.1	0.3 ± 0.0*	0.5 ± 0.0	0.8 ± 0.0	n.a
PSBE	ATCG00580.1	0.3 ± 0.1*	0.5 ± 0.1	0.9 ± 0.1	0.8 ± 0.2	0.7 ± 0.2	2.2 ± 0.3	n.a
PSAC	ATCG01060.1	0.2 ± 0.1*	0.3 ± 0.1	1.0 ± 0.1	0.2 ± 0.0*	0.3 ± 0.0	1.0 ± 0.0	n.a

Notes: See notes of Table 1

### Down-regulation of photosystem proteins is associated with over-accumulation of CLPC2 in the *clpc1* mutant

**Fig. 3:**
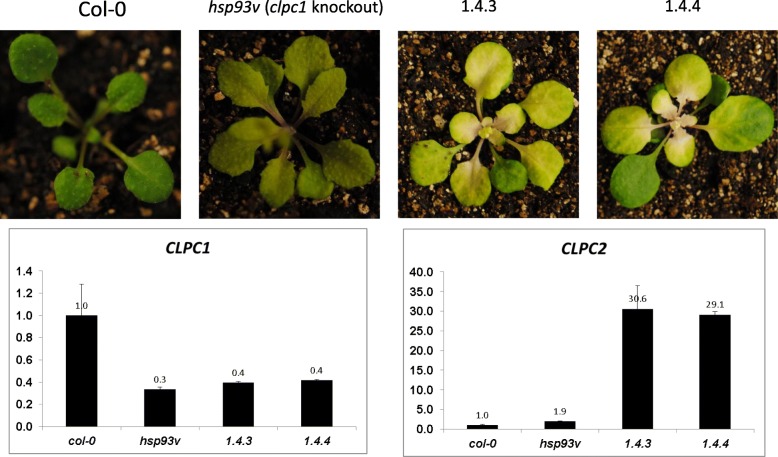
Overexpressing CLPC2 in the *hsp93V/clpc1* mutant causes chlorosis phenotypes under normal light conditions. Seedlings were transferred to soil from MS plates and the pictures were taken 10 days later. *hsp93V, a clpc1* knockout allele in the Col-0 background; 1.4.3 and 1.4.4 are two independent transgenic lines overexpressing *CLPC2* in the *hsp93v/clpc1* knockout mutant background

It has been suggested that photosynthesis genes (photogenes) in chloroplasts are transcribed by chloroplast-encoded eubacteria-like RNA polymerases (PEP) [39, 47, 48]. Although PEP subunit proteins (Table 2) as well as the sense transcripts of the photogenes were over-accumulated in the *clpc1* mutant and ΔN (Fig. 2) line, proteins encoded by photogenes were accumulated less in these plants than in the wild type and the full-length CLPC1 complementation line (Table 5b). The observation of reduced accumulation of photosystem proteins also was confirmed in the *clpp3* knock-out line [31]. These results suggest that there are probably mechanisms limiting the accumulation of those proteins even in the absence of components of the CLPRT complex. Interestingly, there is a concurrent accumulation of the CLPC2 protein in the *clpc1* mutant (Table 4a), a phenomenon that was also noted earlier [49]. CLPC2 has been suggested to act antagonistically to FTSH2 (VAR2), a protease involved in photosystem II repair during photoinhibition [50], and thus accelerate photooxidative stress. Accordingly, both the *clpc1* mutant and the ΔN line over-accumulated CLPC2 proteins and had pale green leaves with reduced levels of photosystem proteins. The under accumulation of these photosystem proteins could be due to over accumulation of CLPC2 although we cannot rule out that this could be an indirect effect caused by the *clpc1* mutation. In contrast, the *clpc2* mutant had dark green leaves, and plants over-expressing CLPC2 showed accelerated photooxidative stress and leaf chlorosis (Fig. 3) [50], especially when the seedlings were grown under normal or high light conditions. It was reported that only a subset of plants overexpressing CLPC2 had the leaf chlorosis phenotype [51]. That all the CLPC2-overexpression plants [51] in our hands exhibited chlorosis may be because the seeds we used were from a progenitor with the chlorosis phenotype.

### The PPR protein SVR7 as a direct target of CLPC1

**Fig. 4:**
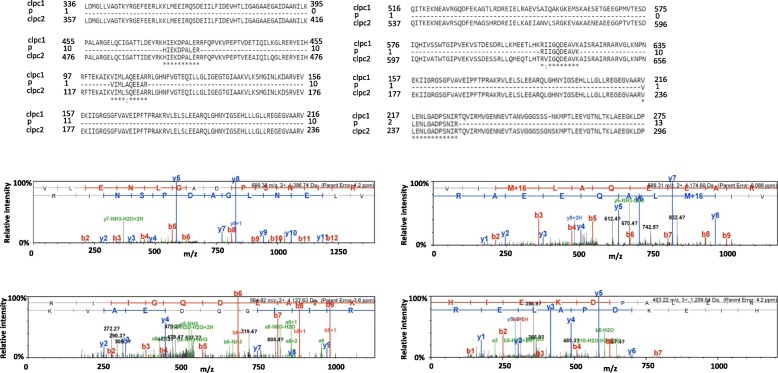
Four unique peptides were identified in a Co-IP experiment using anti-GFP antibody to pull down SVR7-GFP tag. Upper panels: alignment among CLPC1, CLPC2 and the identified peptides (P). Lower panels: Spectra of the four unique peptides

SVR7, a PPR protein, was found to accumulate in the *clpc1* mutant (Table 2). This protein is required for FtsH-mediated chloroplast biogenesis [23] and the accumulation of ATP synthases and their functional transcripts [52]. Its RNA-binding ability and potential involvement in chloroplast RNA processing make us to ask whether SVR7 is a target of CLPC1. To this end, we examined whether SVR7 interacts with CLPC1. We conducted co-immunoprecipitation (Co-IP) assays using GFP-tagged SVR7. Six peptides belonging to CLPCs were identified. Two out of the four unique peptides identified are CLPC1-specific peptides and the other two could be from either CLPC1 and/or CLPC2 since these regions are identical between the two proteins (Fig. 4). These two CLPC1 unique peptides have high Mascot ion score (Additional file 1: Table S3). Since CLPC2 has a far lower expression level than CLPC1 in the wild type background, it is likely that the other two peptides that are common to both proteins are also from CLPC1. Whereas the negative control GFP-tagged AtYAK1 (a cytoplasm localized protein kinase, At5g35980) did not immunoprecipitate with any CLPC proteins, although other chloroplast proteins also were pulled down with the negative control. The results show that SVR7 may be targeted by CLPC1 and mutation in CLPC1 would lead to SVR7 protein accumulation in the *clpc1* mutant. As a result, ATP synthase transcripts were also over-accumulated in the mutant (Additional file 1: Figure S2).

## Discussion

We used iTRAQ-based quantitative proteomics technology to investigate the role of CLPC1 in chloroplast protein homeostasis using the wild type, the *clpc1* mutant, and ΔN and the full-length CLPC1 complementation lines. Our results are consistent with previous data that were obtained with other technologies such as immunoblot [11], gel-based protein excision and MS/MS analysis [32] (Table 1a and b), demonstrating the reliability of our quantitative proteomics data. In addition to using the *clpc1* mutant and wild type, we also included ΔN (a N-terminal deleted complementary line) and CP (full-length CLPC1 complementary line) to better understand CLPC1 and its N-terminal functions in chloroplast proteome homeostasis. In total, we identified more than 800 chloroplast proteins, among which are the proteins that have previously been reported to be mis-regulated by the *clpc1* mutation.

CLPC1 is recognized for its functions as a chaperone in precursor protein import as well as in chloroplast protein degradation [11, 13, 29]. CLPC1 participates in these processes by acting as a component in the TIC complex [53] and the CLP protease complex [10, 31, 54, 55]. Nonetheless, little is known about CLPC1’s role in chloroplast RNA homeostasis, although there has been speculation that CLPC1 might play a role in chloroplast gene expression [56]. In a previous study, although no significant up-regulation of RH3, SVR7, rpoC2, and the PPR proteins AT5G46580 and pTAC2 in the *clpc1* single mutant, these RNA metabolism-related proteins were found to be significantly accumulated in the *clpc1 clps1* double mutant [28]. Perhaps due to differences in detection methods used or experimental conditions, our proteomics experiments showed that CLPC1 regulated the level of PEP proteins (rpoA, rpoB, rpoC1), PPR proteins (MRL1, SVR7, and MEE40), RNases (RNase J, PROPR1, CSP41B), RNA-binding (CP29, CP33, RPB31, RH3 and others) and RNA modification proteins (RIF10, 16 s rRNA processing protein, and RNA 3′ phosphate cyclase) (Table 2). These chloroplast proteins may control chloroplast RNA biogenesis or stability, and thus affect RNA levels and chloroplast gene expression. Our gene-specific RT-PCR results showed that the over-accumulated RNA-biogenesis and metabolism proteins in the *clpc1* mutant and in ΔN indeed were associated with altered chloroplast RNA levels. Specifically, genes for those over-accumulated proteins were found also to have a higher abundance of their transcripts (Fig. 2, Additional file 1: Figure S1). Surprisingly, some genes with less protein abundance in the *clpc1* mutant and ΔN had more transcripts (Fig. 2, Additional file 1: Figure S1) in the mutant and the ΔN line than in the wild type, and the transcript levels were restored to the wild-type levels in the full-length CLPC1 complementary line. These results showed that the high level of transcripts in the *clpc1* mutant was caused by the deficiency of the wild-type CLPC1 functions.

Originating from prokaryotic photosynthetic bacteria via endosymbiosis [1], chloroplasts still retain certain prokaryotic genome traits. Most genes in the chloroplast genome are transcribed in polycistronic clusters [2]. The abundant PPR proteins and other RNA-binding proteins in the *clpc1* mutant may prevent RNases from degrading their bound RNAs. Whereas the differential accumulation of sense RNAs in chloroplasts is more or less consistent with the over accumulation of certain proteins in the *clpc1* mutant, there are other proteins with a decreased accumulation in the *clpc1* mutant. The disparity in the reduced protein levels of these proteins despite their higher transcript levels may partly result from increased proteolysis of these particular proteins or from the lack of functional ribosomal components as reported [46]. Our proteomics data showed that the chloroplasts of the *clpc1* mutant (and ΔN) accumulated more proteases than those of the wild type (Additional file 1: Table S1). It is known that certain proteins such as photosystem proteins are degraded by proteases in an ATP- or GTP-independent manner [46]. Indeed, our proteomics data indicated that nearly all photosystem proteins accumulated less in the *clpc1* mutant and in the ΔN line (Table 5b). Similarly, ClpR4 (a component of ClpPR protease complex) shortage also caused the decreasing of PSI core and PSII core proteins [51]. These data suggest a compensatory CLPR protease-independent proteolysis of these proteins.

The N-terminus of CLPC1 was suggested to have important roles in membrane association [29] and also to interact with CLPS [28] in selecting some targets. Indeed the N-terminus-deleted CLPC1 failed to complement the *clpc1* mutant’s morphological phenotypes (Fig. 1) and its molecular phenotypes except for the restored normal levels of some proteins (Additional file 1), demonstrating the importance of the N-terminus to CLPC1 function.

The CLP protease complex includes not only proteolytic subunits (CLPPs) and the non-catalytic subunit CLPR but also CLPC/D chaperones [33, 51], as well as the CLPS adaptor protein [28]. A recent report showed that CLPS interacts with CLPC1 and CLPC2 at their N-termini and that the CLPS level was up-regulated in the *clpc1* mutant [28]. CLPS has been suggested to be a crucial factor in the N-end rule pathway. In this proteolytic pathway, the N-terminal residues of short-lived proteins are recognized by recognition components (N-recognins) as essential components of degrons [57]. CLPS binds directly to N-terminal destabilizing residues (N-degron) to deliver substrates to ClpAP for degradation (CLPA in *E. coli* is equivalent to CLPC1) [58]. Using affinity chromatography, Nishimura et al. showed that CLPC1 plays a role in chloroplast protein homeostasis, and its interaction with CLPS is important for some CLP protease substrate selection and degradation [28]. Interestingly, we found that CLPC1 also directly interacts with the PPR protein SVR7 (Fig. 4), consistent with CLPC1’s role as a chaperone in mediating the degradation of substrate proteins. It should be mentioned that, in our Co-IP assays, 4 of the peptides from the pull-downed protein(s) matched to CLPC1, while other 2 matched to sequences that are shared by both CLPC1 and CLPC2. Therefore, we cannot rule out the possibility that CLPC2 also interacts with SVR7.

**Fig. 5:**
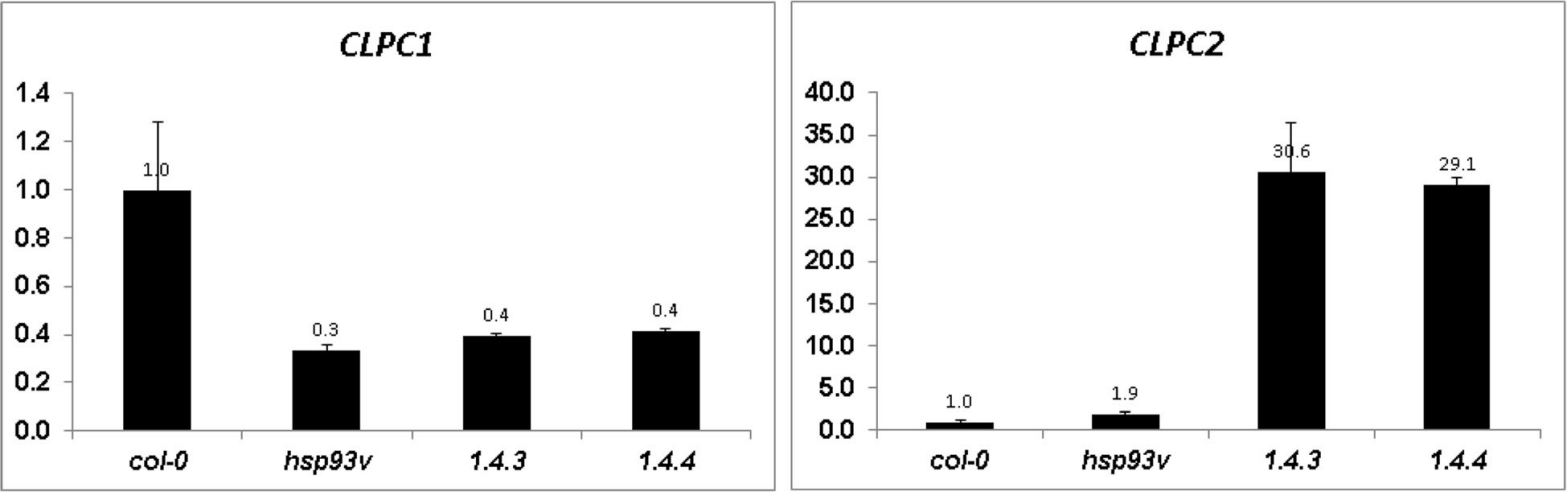
The expression level of *CLPC1* and *CLPC2* in seedlings of the indicated genotypes relative to that in the wild type plants. Shown are means and SD from 3 replicates. qRT-PCR was conducted using gene-specific primers (Additional file 1: Table S2) normalized against the expression of the *ACTIN2* Gene. WS, the wild type; *clpc1*, the *clpc1* mutant; ΔN, *clpc1* expressing N-terminus-truncated CLPC1; CP, *clpc1* expressing the full-length wild-type CLPC1

ClpC1 and ClpC2 share approximately 93% amino acid sequence similarity [10]. While *clpc1 clpc2* double knock-out lines are inviable, the *clpc1* knock-out line has pale green leaves, growth retardation, low photosynthesis activity [11, 12], and increased CLPC2 protein accumulation (Table 2), as well as increased *CLPC2* transcript accumulation (Fig. 5). Overexpressing *CLPC2* in the *clpc1* mutant background complemented *clpc1* mutant chlorosis phenotype at the 1–2 week seedling stage [59]. However, when these CLPC2 overexpressed lines were transferred to soil and grew under normal light conditions further for more than 10 days, all the younger leaves exhibited a strong chlorosis phenotype (Fig. 4), similarly as previously reported [50]. Furthermore, the CLPC2 overexpressing line in *clpc1* mutant background also could greatly restore the chloroplast RNA level, and rescue the RNA accumulation phenotype in the *clpc1* mutant (Additional file 1: Figure S2), indicating their functional similarity and redundancy of these two proteins.

## Conclusions

**Fig. 6:**
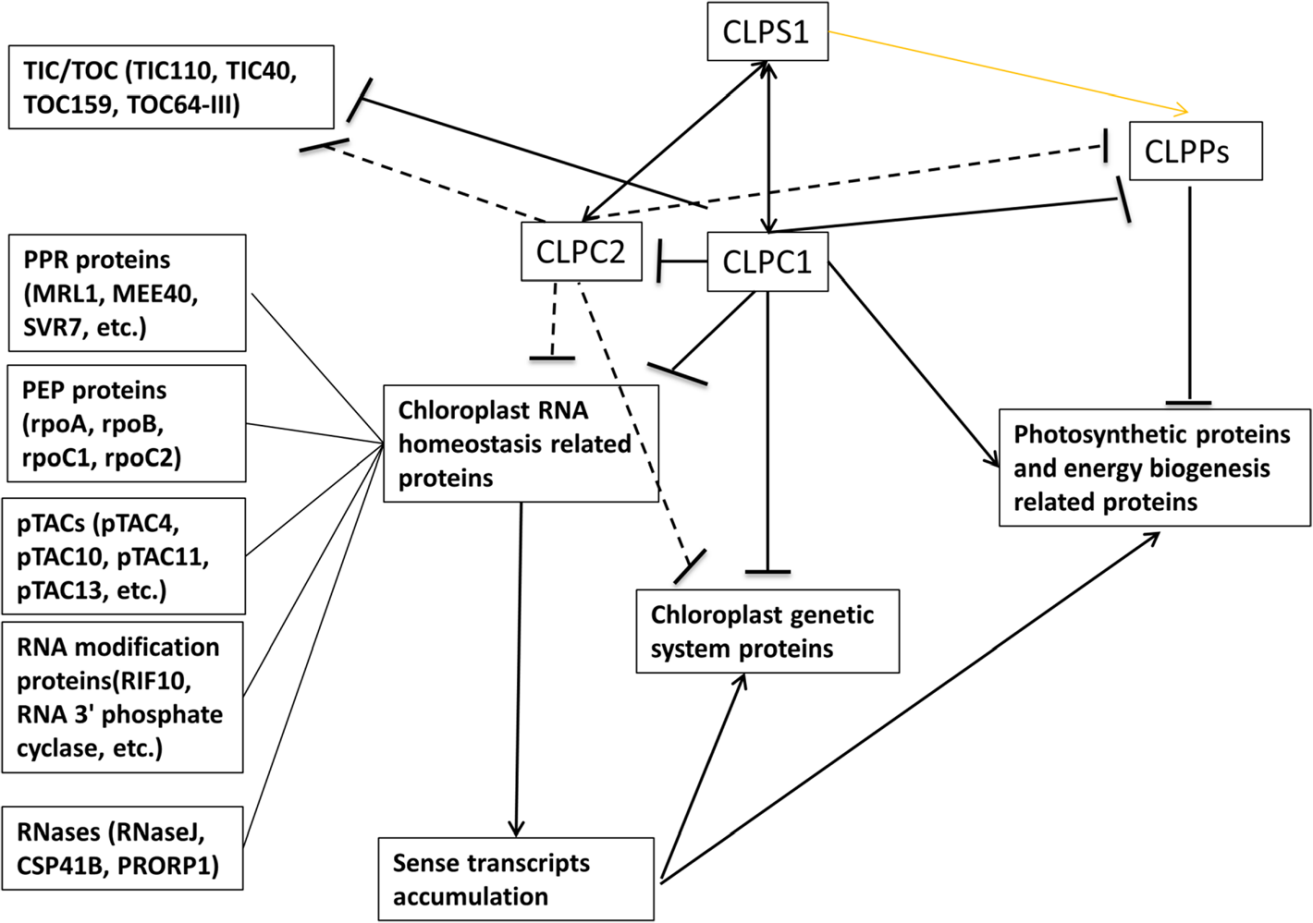
CLPC1’s possible roles in directly or indirectly mediating chloroplast protein and RNA homeostasis. Arrows indicate positive regulation of the abundance of the indicated proteins or RNAs; Bars indicate negative regulation of the abundance of the indicated proteins or RNAs, and double arrows indicate interaction. Solid lines represent regulation supported by experimental evidence; dashed lines denote hypothetical regulation

Based on our study and previous reports, the role of CLPC1 in chloroplast proteome homeostasis can be summarized as follows (Fig. 6). CLPC1 prevents over-accumulation of chloroplast proteins related to RNA homeostasis (such as PPR proteins, PEP proteins, pTACs proteins, RNA modification proteins and RNases), chloroplast genetic system proteins and components of CLPPs as well as pre-protein importing (TIC40, TOC159, TOC64-III etc.) or quality surveillance (TIC110) related proteins. However, it promotes accumulation of CLPS1 and proteins in photosynthetic and energy biogenesis. For target selection, CLPS might guide CLPC1 to its substrates via the N-end rule. While CLPC2 can partly compensate for CLPC1 when CLPC1 is unavailable, CLPC1 can prevent over accumulation of CLPC2.

## Methods

### Plant materials

The wild-type Arabidopsis (WS ecotype), *clpc1* mutant (WS background), and the ΔN (N-terminal deleted CLPC1 complementary line) and full-length CLPC1 complementation (CP) lines (with the *CLPC1* genes driven by the cauliflower mosaic virus *35S* promoter) were described previously [29]. The *hsp93v* (*clpc1*, sail_873_G11) was from the Arabidopsis Biological Resource Center, 1.4.3 (CLPC2 overexpressing in the *clpc1* knockout background), 1.4.4 (CLPC2 overexpressing in the *clpc1* knockout background) were from Dr. Paul Jarvis. Seeds were sterilized with 50% bleach with 0.01% Trion X-100, and then washed 5 times with sterilized double distilled H_2_O. The sterilized seeds were placed onto a half-strength Murashige and Skoog (MS) salt medium, supplemented with 3% sucrose and 0.6% agar. After 4 days of cold stratification, plates were incubated at 22 °C under constant white light for seed germination and seedling growth. Around 14 days old seedlings were documented and transplanted to soil and further grew for 2 to 4 weeks under long-day (16 h light/ 8 h dark) conditions before chloroplast harvesting. Two independent proteomics experiments were performed. The first set used 4-week-old seedlings and the second set used 2-week-old seedlings (with two biological replicates). These growth periods correspond to the period when significant expression of *CLPC1* has been documented.

### Chloroplast isolation

Chloroplasts were isolated as described by Wilson et al. (2011) [60]. Briefly, plants were incubated in the dark for 12 h before chloroplast isolation. Large rosette leaves were cut and immediately immersed in a protoplast buffer (20 mM MES-KOH pH 5.2, 400 mM Sorbitol, 0.5 mM CaCl2 with 1.5% cellulase and 0.4% macroenzyme, 0.1% BSA) for 3 h. The protoplasts were then filtered with a 70-μm cell strainer and centrifuged. The materials were then resuspended/rinsed in 5 ml protoplast buffer by gentle swirling and centrifuged for 2 min at 100 *g* at 4 °C. The pellets were resuspended in 5 ml of buffer protoplast breaking buffer (20 mM Tricine-KOH pH 8.4, 300 mM Sorbitol, 5 mM EDTA, 5 mM EGTA, 10 mM NaHCO3, and 0.1% BSA). The suspension was passed through a 20-μm mesh and collected onto a chilled 40/85 percoll step column. The column was then centrifuged in a swinging rotor for 10 min at 2500 *g* at 4 °C with the brake off. The lower band was harvested using a pipette and transferred to a 50 ml tube and diluted with 40–45 ml of HEPES-sorbitol buffer (50 mM HEPES-KOH, pH 8.0, 330 mM Sorbitol). The sample was centrifuged for 5 min at 700 *g* at 4 °C and re-suspended in 200 μl of HEPES-sorbitol buffer (pH 8.0).

### RT-PCR

One μg of total RNAs from each of WS, *clpc1*, ΔN, and the full-length CLPC1 complementation line was used for gene-specific reverse transcription using the Superscript III first strand synthesis kit (Invitrogen). We used the reverse primers for 49 chloroplast and nuclear (*CLPC1* and *CLPC2*) genes and one reverse primer for the *ACTIN2* gene in quantitative PCR (qPCR) for first strand cDNA synthesis (100 μM of each reverse primer were mixed, which gave a final concentration of 2 μM for each of the 50 reverse primers). The reverse-transcribed cDNA was first used for PCR to check whether the expected fragment was obtained and then used for quantitative RT-PCR to assess the transcript abundance. The primers used in the study are listed in Additional file 1: Table S2.

### Co-immunoprecipitation (co-IP) experiments

Two-week-old Arabidopsis seedlings (ecotype Col-0) harboring the *35S::SVR7-GFP* transgene were digested with protoplast buffer (20 mM MES-KOH pH 5.2, 400 mM Sorbitol, 0.5 mM CaCl2 with 1.5% cellulase and 0.4% macroenzyme, 0.1% BSA) for 3 h. Seedlings expressing 35S promoter driven YAK1 tagged with GFP at its C-terminus (*35S::YAK1-GFP*) were used as a control for the Co-IP. The digestion solution was filtered with a 70-μm cell strainer, and centrifuge at 100 x *g* for two minutes to pellet the protoplasts. After being washed three times with ice-cold PBS buffer (137 mM NaCl, 2.7 mM KCl, 10 mM Na2HPO4, and 1.8 mM KH2PO4, pH 7.4), 200 μl of lysis buffer (10 mM Tris/Cl pH 7.5, 150 mM NaCl, 0.5 mM EDTA, 0.5% NP40, 1 × protease inhibitor cocktail, and 1 mM PMSF) were added and the pellet was re-suspended by extensive pipetting. The sample was incubated on ice for 30 min with extensive pipetting every ten minutes and spun for 10 min at 4 °C at 16100 x *g*. The supernatant was transferred to a pre-cooled tube, and the volume was adjusted with dilution buffer (10 mM Tris/Cl pH 7.5, 150 mM NaCl, 0.5 mM EDTA, 1 × protease inhibitor cocktail, and 1 mM PMSF) to 1 ml. This cell lysate was added to equilibrated GFP-Trap_A beads and incubated under constant mixing for 2 h at room temperature. The beads were washed three times with washing buffer (10 mM Tris/Cl pH 7.5, 150 mM NaCl, 0.5 mM EDTA, 1× protease inhibitor cocktail, and 1 mM PMSF), and, after the first washing, the NaCl concentration was increased to 500 mM. The bound proteins were eluted by adding 50 μl 0.2 M glycine (pH 2.5) and incubated for 30 s under constant mixing followed by centrifugation. The supernatant was transferred to a new tube, and 5 μl 1 M Tris base (pH 10.4) were added for neutralization. The sample was subjected to electrophoresis in 2 x SDS sample buffer for 12 min and the gel was excised for in-gel digestion and LC-MS/MS analysis.

### Peptide preparation, iTRAQ labeling and strong Cation exchange fractionation

Two hundred μl of chloroplasts in HEPES-sorbitol buffer (pH 8.0) were sonicated three times each for ten seconds at two minutes intervals using Qsonica LLC XL-2000 with the power output set at 8. Then the solution was acetone-precipitated (acetone:sample = 5:1 *v*/v) overnight at − 20 °C. The protein pellet was recovered by centrifugation at 12,000 *g* at 4 °C for 10 min, rinsed with cold acetone three times, and air-dried. The protein pellet was then resuspended in the buffer containing SDS-PAGE sample buffer without dye. The protein concentration was determined using a 2D Quant kit (GE Healthcare). Approximately 100 μg proteins of each sample were then loaded into a 10% SDS-PAGE gel and run for 25 min to separate proteins from other non-proteins/small molecules. After Coommassie blue staining, the total proteins were used for in-gel digestion with trypsin. The eluted peptides were dried using a Speedvac (Eppendorf, Hamburg, Germany) and labeled with iTRAQ reagents (Applied Biosystems, Framingham, MA, USA) according to the manufacturer’s protocol. Briefly, peptides were reconstituted in 30 μl of dissolution buffer (0.5 M TEAB) and mixed with 70 μl of ethanol-suspended iTRAQ reagents (one iTRAQ reporter tag per sample). Labeling reactions were carried out at room temperature for 60 min before all four samples were mixed in a single tube and dried using a SpeedVac. Strong cation exchange fractionation of the combined peptide mixture was carried out as previously described [61, 62]. Ten fractions were finally obtained, desalted, and dried.

### Mass spectrometric analysis using LTQ-Orbitrap

Each dried fraction was reconstituted in 20 μl of 0.1% formic acid and acetonitrile just before mass spectrometric analysis. The labeled sample was analyzed three times on an LTQ-Orbitrap Velos (Thermo Scientific, Germany) coupled with an Easy-nLC (Thermo Scientific). Five microliters of the sample were injected for each analysis and concentrated in a preconditioned column (0.3 × 50 mm) packed with C18 AQ (5 μm particles, 200 Å pore size) (Bruker-Michrom, Auburn, CA, USA). The peptide separation was performed in a preconditioned capillary column (0.1 × 150 mm, with C18 AQ of 3 μm particles and 200 Å pore size (Bruker-Michrom)). The peptide was separated using a 60-min gradient comprised of 35 min of 0–35% mobile phase B (0.1% formic acid in acetonitrile (ACN)), 10 min of 35–80% B, and 15 min of 80% B. The total flow rate of the gradient was set at 400 nl/min. The sample was introduced into the LTQ-Orbitrap through a Nanospray Flex (Thermo Scientific) with an electrospray potential of 1.5 kV. The ion transfer tube temperature was set at 160 °C. The LTQ-Orbitrap was set to perform data acquisition in the positive ion mode. A full MS scan (350–1600 m/z range) was acquired in the Orbitrap at a resolution of 30,000 (at 400 m/z) in the profile mode with a maximum ion accumulation time of 1 s and a target value of 1 × e6. Charge state screening for the precursor ion was activated. The six most intense ions above a 1000-count threshold and carrying multiple charges were selected for a paralleled fragmentation (MS/MS) in the collision-induced dissociation (CID) in the linear ion trap and the higher energy collision dissociation (HCD) in the Orbitrap. Dynamic exclusion for both CID and HCD fragmentation was activated with a repeat count of 2, a repeat duration of 30 s, an exclusion duration of 45 s, and ± 5 ppm mass tolerance. The additional CID settings included a maximum ion accumulation time of 200 ms for MS/MS spectrum collection, a target value of 1 × e4, a normalized collision energy at 35%, an activation Q at 0.25, an isolation width of 3.0 and an activation time of 10 ms. The HCD settings included a full scan with the Orbitrap at a resolution of 7500 (at 400 m/z) in a centroid mode, a maximum ion accumulation time of 200 ms for MS/MS spectrum collection, a target value of 5 × e4, a normalized collision energy at 40%, an isolation width of 3.0 and an activation time of 0.1 ms.

### Mass spectrometric data analysis

The MS raw data were processed using the Proteome Discoverer software (version 1.2, Thermo Scientific) to extract mascot generic files (mgf) from HCD and CID spectra separately. The four iTRAQ reporter ions had m/z of 114.112, 115.108, 116.116 and 117.115 respectively. These reporter ions and their intensities for each parent ion were extracted from the HCD mgf files. The mass tolerance for the extraction was set at 10 mDa. The extracted reporter ions were inserted back into both HCD and CID mgf files, whereas their original iTRAQ mass region (114.0–117.5) was cleared. The modified HCD and CID mgf files were analyzed using Mascot (Matrix Science, London, UK; version 2.4.0) [63], which searched the concatenated target-decoy Arabidopsis protein database TAIR10 [30] with common contaminants (71,248 entries). The enzyme limits were set at full tryptic cleavage at both ends, and a maximum of one missed cleavage was allowed. The mass tolerances were set to 10 ppm for the peptide precursors and 0.5 Da for the fragment ions. Variable modifications for the search included iTRAQ (4-plex, 144.10) at tyrosine and oxidation (+ 15.99) at methionine. The fixed modifications were carbamidomethylation (57.02) at cysteine and iTRAQ (4-plex) reagent labeling at N-terminal and lysine.

The mascot search results were exported in *csv* files and only peptides with expectation value less than 0.05 were included and used for quantitation. Peptide quantification was normalized based on the total intensity of the assigned mass spectrum according to Mascot searching result. The protein ratios were calculated accordingly from the weighted sums of the normalized peptide intensity.

## Additional file

Additional file 1: **Table S1.** Proteases accumulation in seedlings of different genotypes. **Table S2.** Primers used in the study. **Table S3.** Peptides identified of CLPC1 in SVR7-GFP CO-IP experiment. **Figure S1.** Relative expression levels of sense transcripts in the *clpc1* mutant and its complementation lines. **Figure S2.** Over-expressing CLPC2 in *clpc1* mutant partially or fully restored the chloroplast RNA level. **Figure S3.** Schematic diagram to show the plant materials we used. **Supplementary dataset 1.** Spectral examples of 4 proteins from our iTRAQ-based proteomics analysis. (PDF 2601 kb)

## Abbreviations

ACN: Acetonitrile
CID: the collision-induced dissociation
CLPC: ATP-dependent Clp protease chaperone subunit C1
CLPD: ATP-dependent Clp protease chaperone subunit D
CLPP: ATP-dependent Clp protease proteolytic subunit
Co-IP: Co-immunoprecipitation
CP: full-length CLPC1 complementation (CP) lines
CP29: CHLOROPLAST RNA-BINDING PROTEIN 29
EDTA: Ethylenediaminetetraacetic acid
EF-Ts: translation elongation factor thermo stable
EGTA: Ethylene glycol-bis(β-aminoethyl ether)-N,N,N′,N′-tetraacetic acid
HEPES: 4-(2-hydroxyethyl)-1-piperazineethanesulfonic acid
HSP70: HEAT SHOCK PROTEIN 70
iTRAQ: Isobaric tag for relative and absolute quantitation
LC-MS/MS: Liquid chromatography tandem mass spectrometry
MES: 2-(N-morpholino)ethanesulfonic acid
mgf: mascot generic files
MS: Murashige and Skoog
NEP: Nucleus-Encoded RNA Polymerase
PEP: Plastid-Encoded Polymerase
PMSF: Phenylmethanesulfonylfluoride
PPR: Pentatricopeptide repeat proteins
PTACs: Plastid transcriptionally active chromosome proteins
qPCR: quantitative PCR
RH3: RNA helicase
RT-PCR: Reverse transcription polymerase chain reaction
SVR7: SUPPRESSOR OF VARIEGATION 7
TIC: Translocon at the inner envelope membrane of chloroplasts
TOC: Translocon at the outer envelope membrane of chloroplasts
ΔN: N-terminal deleted CLPC1 lines
