# Supplementary material for: The caseinolytic protease complex component CLPC1 in Arabidopsis maintains proteome and RNA homeostasis in chloroplasts
Source: BMC Plant Biol. 2018 Sep 12;18:192. doi: 10.1186/s12870-018-1396-0 (PMC6136230; doi:10.1186/s12870-018-1396-0)

The caseinolytic protease complex component CLPC1 in Arabidopsis maintains proteome and RNA homeostasis in chloroplasts

ShouDong Zhang[1,2¶], Huoming Zhang[2], Yiji Xia[1,3¶], Liming Xiong[2¶]

[1] Department of Biology, Hong Kong Baptist University, Hong Kong; [2] Division of Biological and Environmental Sciences and Technology, King Abdullah University of Science and Technology (KAUST), Thuwal 23955-6900, Saudi Arabia; [3]Partner State Key Laboratory of Agrobiotechnology, Chinese University of Hong Kong, Hong Kong

Supplementary data

Table S1. Proteases accumulation in seedlings of different genotypes

		1st batch			2nd batch (2 biological replicates)			Nishimura et al (2013)
proteases		4-week-old (LD)			2-week-old (LD)			6-week-old (SD)
symbol	accession	*clpc1/WS*	*ΔN/WS*	*CP/WS*	*clpc1/WS*	*ΔN/WS*	*CP/WS*	*clpc1-1/wt*
CLPP1	ATCG00670.1	4.7±0.3	3.2±0.1	1.0±0.2	1.5±0.0	1.2±0.2	1.0±0.0	1.2
ClpR3	AT1G09130.1	4.6±1.1	2.5±0.6	0.7±0.1	1.8±0.0	1.4±0.1	0.7±0.2	1.4
ClpT1	AT4G25370.1	4.4±0.3	2.8±0.2	0.9±0.1	1.9±0.7	1.2±0.4	1.0±0.0	2.1
CLPR1	AT1G49970.1	4.1±1.5	2.3±0.5	0.9±0.1	2.3±0.9	1.5±0.4	0.7±0.0	1.9
CLPC2	AT3G48870.2	4.0±1.1	3.1±0.5	1.4±0.1	1.5±0.1	1.7±0.0	0.8±0.0	2.5
CLPR2	AT1G12410.1	3.8±0.3	2.7±0.1	0.8±0.1	2.4±0.7	1.5±0.4	0.8±0.1	2.1
CLPP4	AT5G45390.1	3.8±0.2	2.6±0.2	0.7±0.1	2.0±0.3	1.6±0.3	0.7±0.1	1.3
CLPP6	AT1G11750.1	3.4±0.3	2.9±0.3	0.9±0.1	1.7±0.5	1.7±0.3	0.7±0.1	3.6
CLPP3	AT1G66670.1	3.3±0.3	2.6±0.2	0.9±0.2	1.5±0.2	1.3±0.1	0.7±0.3	1.0
CLPR4	AT4G17040.1	3.1±1.0	2.0±0.4	0.9±0.1	2.0±0.2	1.6±0.1	0.7±0.2	1.5
ClpT2	AT4G12060.1	3.0±0.2	2.1±0.1	0.9±0.1	1.9±0.2	1.5±0.1	1.1±0.1	1.4
CLPB3	AT5G15450.1	2.3±0.9	1.6±0.2	1.1±0.1	2.3±0.3	1.5±0.1	0.7±0.0	3.4
PREP1	AT3G19170.1	4.0±0.5	2.5±0.2	0.9±0.0	1.7±0.1	1.7±0.1	0.6±0.0	1.8
DegP2	AT2G47940.1	3.6±1.1	2.7±0.5	1.2±0.2	1.3±0.2	1.4±0.2	1.0±0.3	2.5
LON	AT1G75460.1	2.5±0.0	2.7±0.1	1.1±0.0	3.0±0.1	2.3±0.1	1.3±0.1	n.a
FTSH12	AT1G79560.1	2.0±0.1	2.0±0.2	0.9±0.0	1.5±0.1	1.9±0.0	1.1±0.0	1.2
AraSP	AT2G32480.1	1.4±0.4	1.2±0.2	1.0±0.0	2.0±0.1	1.6±0.2	1.1±0.1	1.4
DegP1	AT3G27925.1	0.7±0.1	0.9±0.0	1.0±0.0	0.8±0.0	0.8±0.0	1.2±0.1	n.a

Notes:

1. WS: wild type; *clpc1*: the *clpc1* mutant; ΔN, N-terminal (1-93 amino acid) deleted CLPC1 complementary line; CP, full-length CLPC1 complementary line, LD, long-day; SD, short-day.
2. The first batch dataset was from 4-week-old seedlings with three technical replicates. The second batch was from 2-week-old seedlings with two biological replicates and each biological replicate included 3 technical replicates.
3. Data are means and standard errors of protein abundance relative to the wild type (WS).
4. Data from Nishimura et al (2013) in [27].

Table S2. Primers used in the study.

CLPC1-F	CAATCGACTCCACCGTCTTT
CLPC1-R	TCCCATAAACCCTTGCATTCT
CLPC2-F	GAGGGTGTGTGATGAAGATGAG
CLPC1-R	TGTCAGAAGAGGTGCTTGTAAA
pClpP-F	CGACCCGATGTACAGACAATA
pClpP-R	TAGCGTGAGGGAATGCTATAC
ACT2-F	TGCTTATGTCGCTCTTGACTAC
ACT2-R	CTCTCAGCTCCGATGGTTATG
rbcL-F	GTGTTGGGTTCAAAGCTGGT
rbcL-R	CATCGGGTCCACACAGTTGTC
accD-F	TGTGGATTCAATGCGACAAT
accD-R	TTTTGCGCAGAGTCAATACG
atpA-F	CGGAAATCTTACCTCGACCA
atpA-R	ATGGGTGACGGTTTGATGAT
atpH-F	ATCCACTGGTTTCTGCTGCT
atpH-R	TTCCTTCTGCCTCAGGTTGT
atpI-F	ATTGGCAAATAGGGGGTTTC
atpI-R	GCCGTCGATTGGAATTGTTT
ccsA-F	CACAATAACTGCGCCAAGTG
ccsA-R	AACAGAGCGCCATAGCCTAA
cemA-F	TTTGCCCTGGTTGATCTCTC
cemA-R	TTGGATCGTTTCTTTGTGGA
ndhB-F	CCAGAAGAAGATGCCATTCA
ndhB-R	TCATCAATGGACTCCTGACG
ndhI-F	TTTGCCTGTTGTTGATTGGA
ndhI-R	ATTGGTAAACGACCCAAAGC
petA-F	CAGAGGGCGAATCCATTAAA
petA-R	GCCAAAACAACCGATCCTAA
petB-F	ATTGGGCGGTCAAAATTGTA
petB-R	AGACGGCCGTAAGAAGAGGT
psaA-F	GCCAAGAAATCCTGAATGGA
psaA-R	CATCTTGGAACCAAGCCAAT
psaC-F	GAGCATGCCCTACAGACGTA
psaC-R	CAGGCGGATTCACATCTCTT
psbL-F	CAATCAAATCCGAACGAACA
psbL-R	GAAATAATTCGAAAATAAAACAGCAA
rpL14-F	AGCGGGGCTAGAGAATTGAT
rpL14-R	ACTGCGGCATTGTCATCATA
rpL20-F	TCGGAGGCGTAGAACAAAAC
rpL20-R	CGATGAGCCGAAACTAAAGC
rpoA-F	GCGATGCGAAGAGCTTTACT
rpoA-R	CCAGGACCTTGGACACAAAT
rpoB-F	AAAAAGCACGGATACGGATG
rpoB-R	CTTCTTGAATGCCCCGATTA
rpoC1-F	TCGGATACGAAGATATCAAATGG
rpoC1-R	TTAGTTATGGGCCTAGCAAAAGA

rpoC2-F	ATGGAGCCCGTAAAGGAGTT
rpoC2-R	CGTCTGCTAAGACACGACCA
rpS18-F	CAAGCGATCTTTTCGTAGGC
rpS18-R	AAAGCTCACTCTATTCACCCGTCT
ycf2-F	TAGCCCTCGGTCTATTGGTG
ycf2-R	GGATCCACTTTTTGGGGAAT
ycf3-F	TCCAATACTCAGCGGCTTG
ycf3-R	TTCGGGCATTAGAACGAAAC
ycf5-F	GCGAACAACCGGAGCTATTA
ycf5-R	CCGACATGCGTATTTTTGATT
23s rRNA-F	GGGCGACTGTTTACCAAAAA
23s rRNA-R	TTACCCGACAAGGAATTTCG
16s rRNA-F	CGGTATCTGGGGAATAAGCA
16s rRNA-R	GATTTGACGGCGGACTTAAA
atpB-F	CCGTTTCGTACAAGCAGGAT
atpB-R	CGGGGTCAGTCAAATCATCT
atpE-F	TCCACAAGAAGCTCAGCAAA
atpE-R	GTGTCCGAGCTCGTCTGAG
atpF-F	GCTCCTTCACGCAGTTCTTC
atpF-R	TACTTGGGTCACTGGCCATC
matK-F	ATCCTTTGTTGCCAGAATGC
matK-R	TTTTTCTACGCAAGCGGTCT
ndhA-F	TTGACGCCACAAATTCCAT
ndhA-R	TTAGGTGGTCTGCGAGCTG
ndhD-F	TGGAGAATGGGAATAGATGGAC
ndhD-R	TCCCGAGAAGAAAATGATCCTA
ndhK-F	GCAGTCCGCATATTGGAAAT
ndhK-R	CGTGGGACGATACTGGACTT
petD-F	TCCTTTTGCAACTCCTTTGG
petD-R	CCGCTGGTACTGAAACCATT
psaB-F	GGACCCCACTACTCGTCGTA
psaB-R	ATTGCTAATTGCCCGAAATG
psbA-F	GAGCAGCAATGAATGCGATA
psbA-R	CCTATGGGGTCGCTTCTGTA
psbB-F	CGTGCGACTTTGAAATCTGA
psbB-R	TAGCACCATGCCAAATGTGT
psbC-F	ACTTCCCCACCTAGCCACTT
psbC-R	AGCCCAAAACTGCAGAAGAA
psbD-F	CACAAATCTTTGGGGTTGCT
psbD-R	CCATCCAAGCACGAATACCT
rpL16-F	TGTACGACGTGGTGGAAAA
rpL16-R	GCATTTTTGATGCCGCTATT
rpL22-F	AAAGCTGAGGTGAACCAAGG
rpL22-R	TGTCCCATAGGCCTCCACTA
rpL32-F	CTCGAAAAAGCGTATTCGTAAAA
rpL32-R	TGAAAAAGCTTTCAACGATGTC
rpS2-F	GGGCTCGGTGTCATTATGTT

rpS2-R	TCTTCAACACAGCTGCATCC
rpS4-F	CGATTGGGTATGGCTTTGAC
rpS4-R	ATGGTTTGGCAATTCCTCAG
rpS8-F	CGACCGGGTCTACGAATCTA
rpS8-R	ATTTCTCCGCCGATTCTTTT
rpS14-F	AATCCCCACCGCGTAATAGT
rpS14-R	AACATGCCTGAACCATTTCC

Table S3. Peptides identified of CLPC1 in SVR7-GFP CO-IP experiment

accession No. gene ID	Protein name	probability	percentage of total spectra	unique spectra	total spectra	Peptide sequence	Mascot Ion score	Modifications identified by spectrum
AT5G50920.1	ATHSP93-V	100.00%	0.16%	4	7	HIEKDPALER	18.5	
AT5G50920.1	ATHSP93-V	100.00%	0.16%	4	7	HIEKDPALER	26.6	
AT5G50920.1	ATHSP93-V	100.00%	0.16%	4	7	RIIGQDEAVK	37.4	
AT5G50920.1	ATHSP93-V	100.00%	0.16%	4	7	RIIGQDEAVK	37.5	
AT5G50920.1	ATHSP93-V	100.00%	0.16%	4	7	VIMLAQEEAR	50.2	Oxidation (+16)
AT5G50920.1	ATHSP93-V	100.00%	0.16%	4	7	VLENLGADPSNIR	30.4	
AT5G50920.1	ATHSP93-V	100.00%	0.16%	4	7	VLENLGADPSNIR	48.5	

Figure S1. Relative expression levels of sense transcripts in the *clpc1* mutant and its complementation lines. Shown are means and SD from 3 replicates. qRT-PCR was conducted using gene-specific primers (Table S2) normalized against the expression of the *ACTIN2* gene. WS, the wild type; *clpc1*, the *clpc1* mutant; ΔN-CP, *clpc1* expressing N-terminus-truncated CLPC1; CP, *clpc1* expressing the full-length wild-type CLPC1.

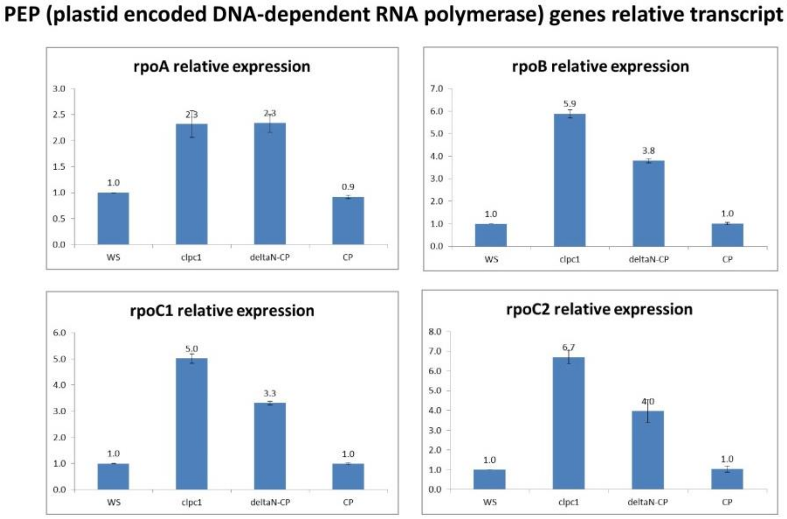

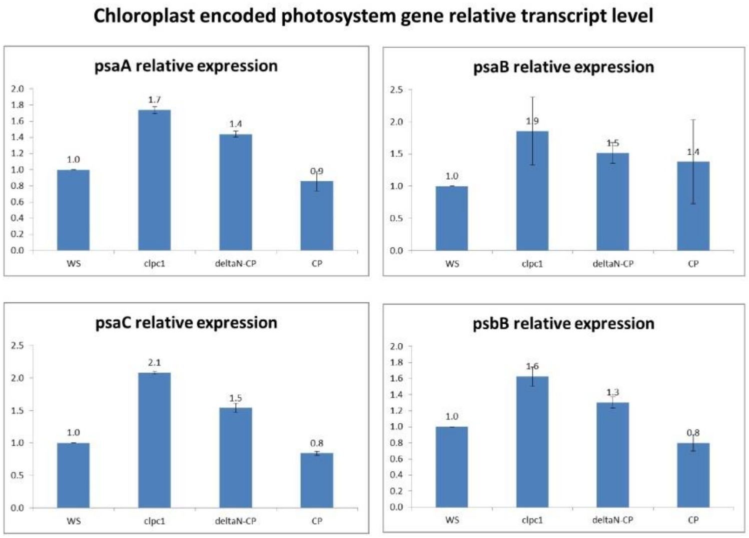

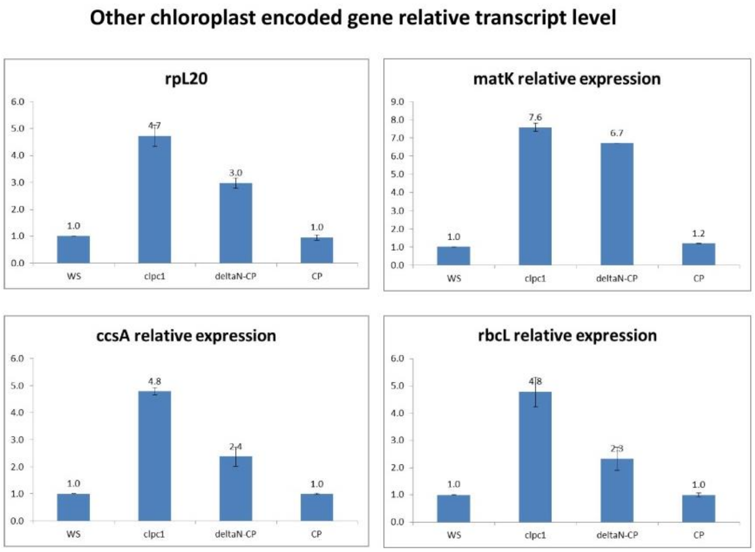

Figure S2. Over-expressing CLPC2 in *clpc1* mutant partially or fully restored the chloroplast RNA level.

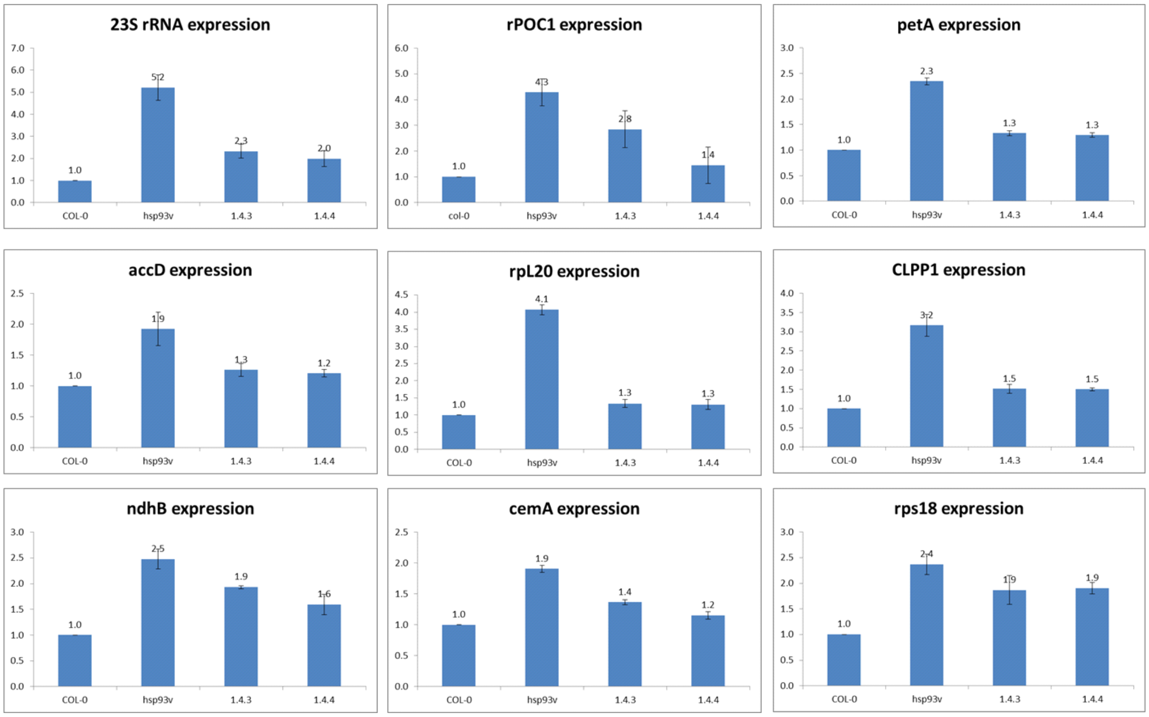

Figure S3. Schematic diagram to show the plant materials we used.

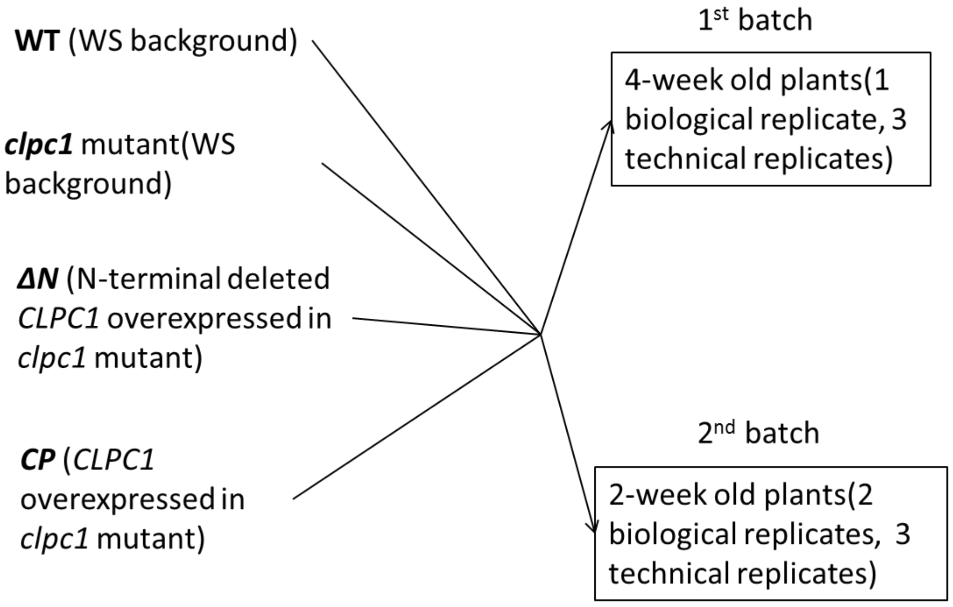

Note: Plant materials(all the plant materials used for proteomics are from Dr. Hsou-min Li (Chu & Li, 2012), and were grown at 21 degree with 16h light and 8h dark)

Supplementary dataset 1. **Spectral examples of 4 proteins from our iTRAQ-based proteomics analysis**

four proteins with each having two peptides and each peptide having two spectra (HCD and CID spectra) were shown below. The protein quantitation value was obtained by the weighted ratios of iTRAQ reporters from all identified peptides that belonged to the protein (enlarged iTRAQ reporter region was shown in the first spectral).

1. AT3G53700.1 [| Symbols: MEE40 | Pentatricopeptide repeat (PPR) superfamily protein |

   a) Matched peptides: **ENQVEEATELAR**

HCD-spectral matched to TAIR10 database with iTRAQ reporter region (zoom in)

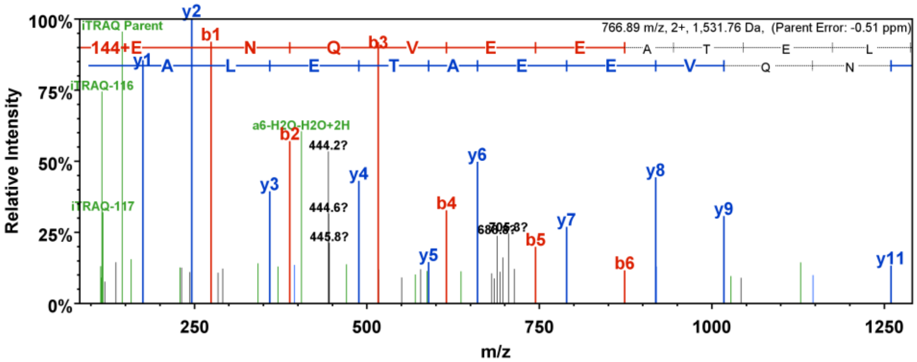

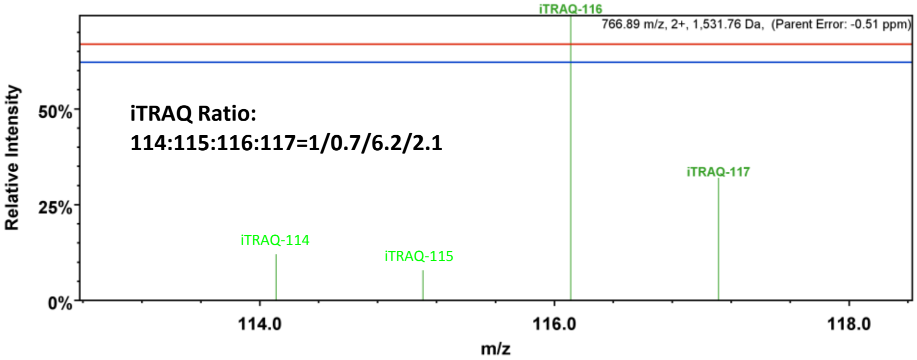

CID-spectral matched to TAIR10 database

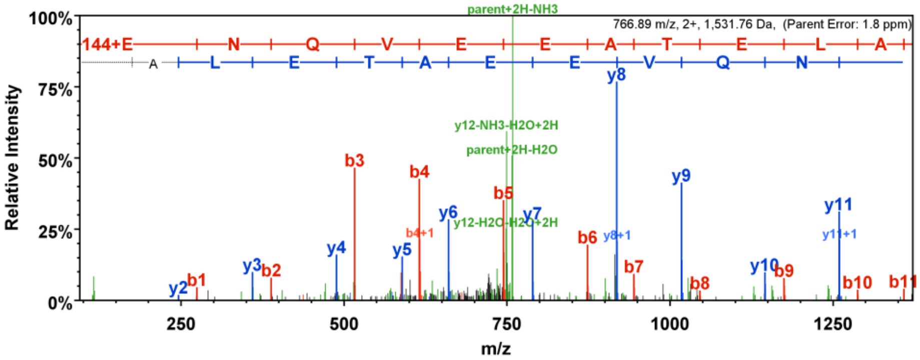

b) Matched peptides: **SQPDDSAALR**

HCD-spectral matched to TAIR10 database

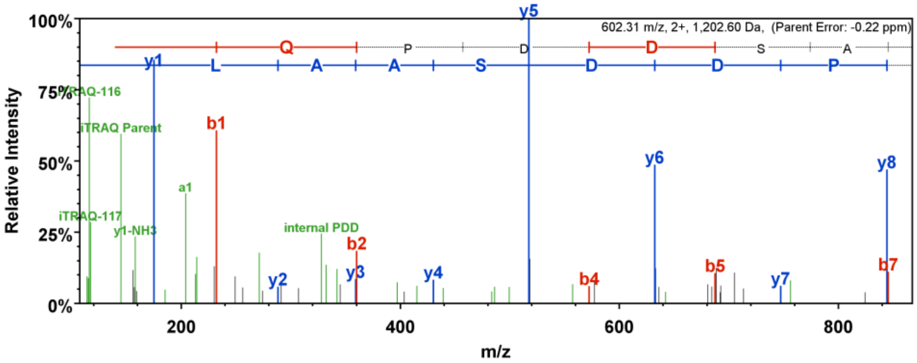

CID-spectral matched to TAIR10 database

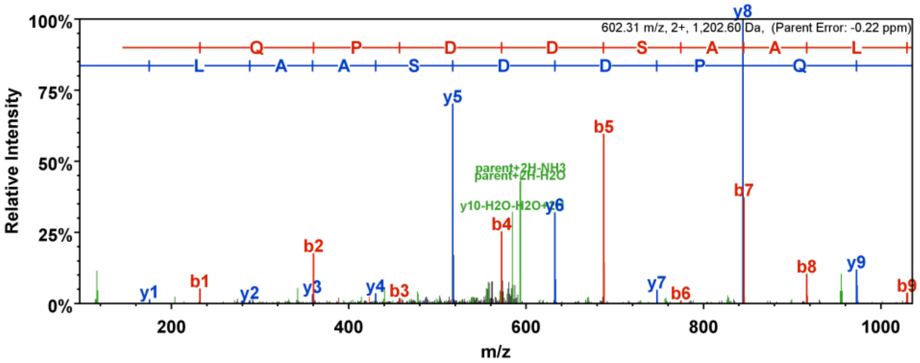

2. AT4G16390.1 [| Symbols: SVR7 | pentatricopeptide (PPR) repeat-containing protein

a) Matched peptides: **EVILYNVTMK**

HCD-spectral matched to TAIR10 database

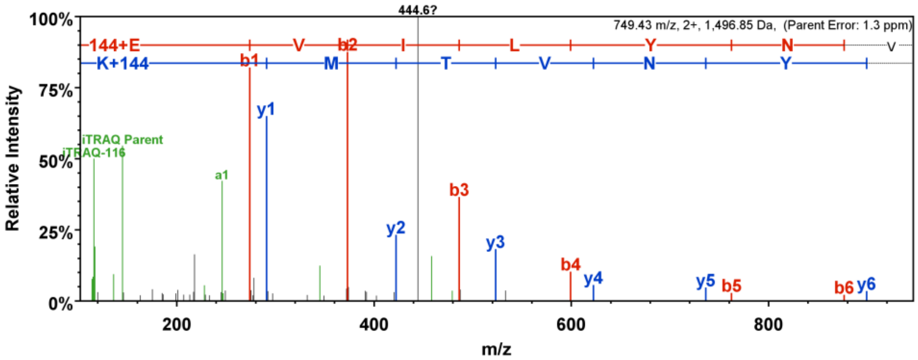

CID-spectral matched to TAIR10 database

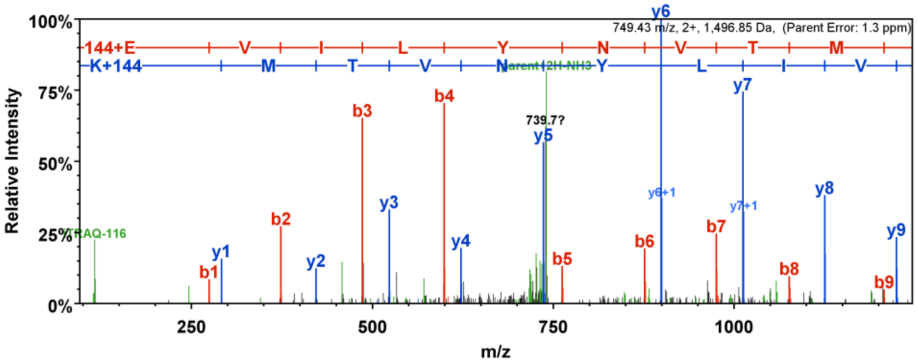

b) Matched peptides: **YGDDALAIYR**

HCD-spectral matched to TAIR10 database

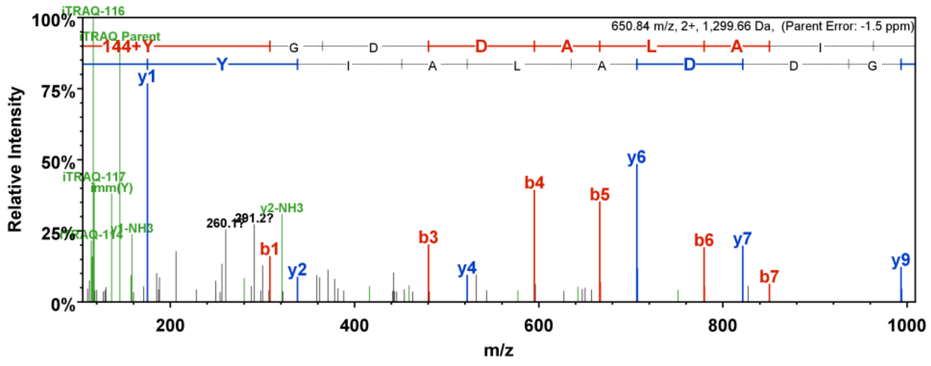

CID-spectral matched to TAIR10 database

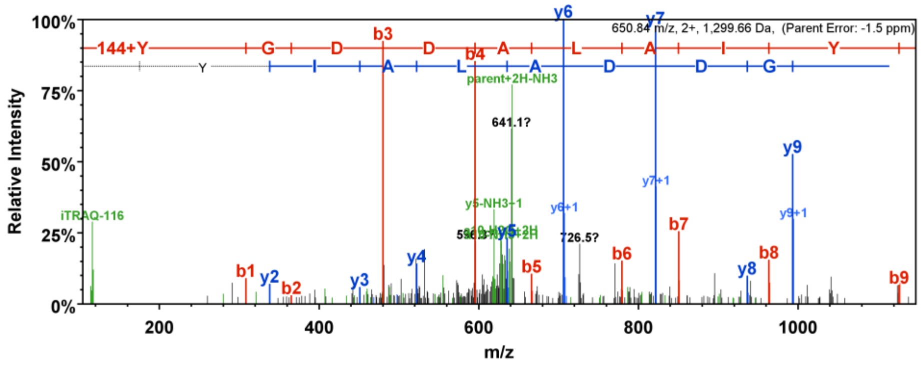

3. ATCG01060.1 [| Symbols: PSAC | iron-sulfur cluster binding;electron carriers;4 iron, 4 sulfur cluster binding

a) Matched peptide: **IYDTCIGCTQCVR**

HCD-spectral matched to TAIR10 database

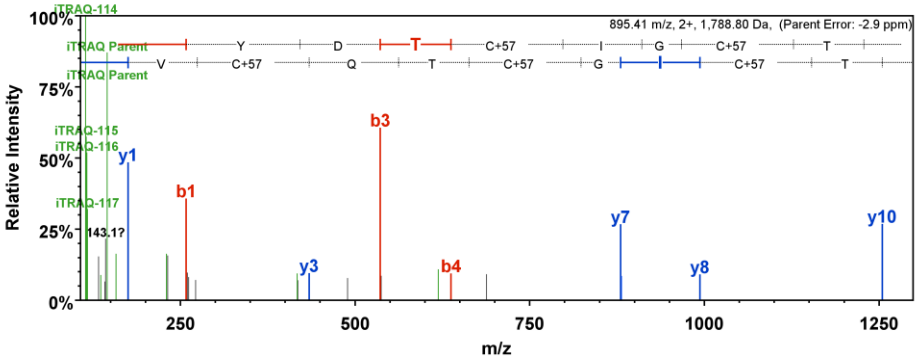

CID-spectral matched to TAIR10 database

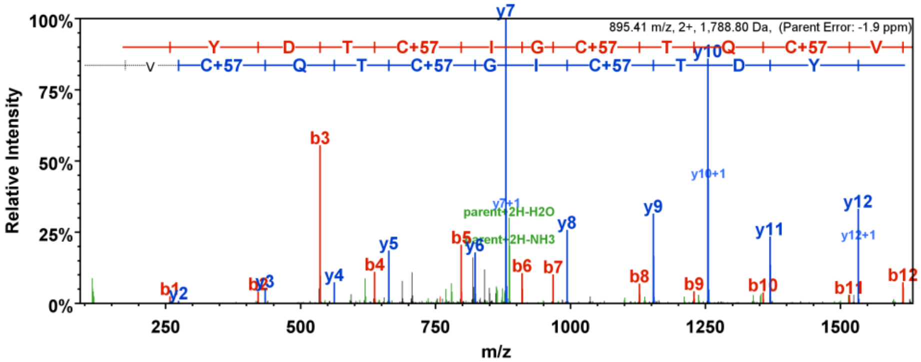

b) Matched peptide: **ACPTDVLEMIPWDGCK**

CID-spectral matched to TAIR10 database (HCD spectral is poor so no match to

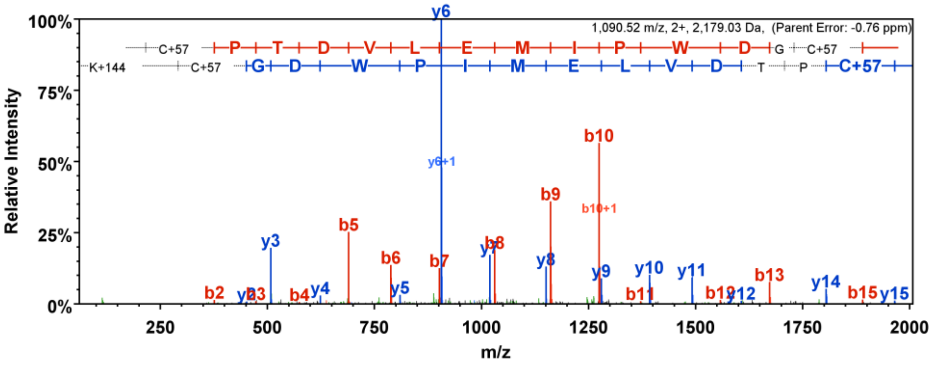

database)

c) Matched peptide: **CESACPTDFLSVR**

HCD-spectral matched to TAIR10 database

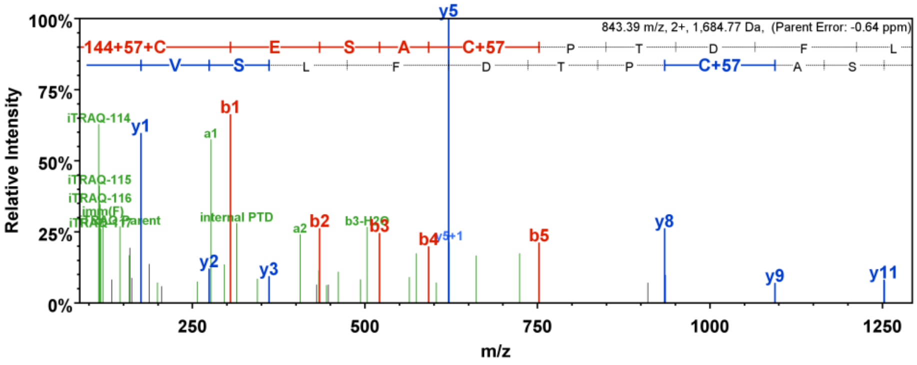

CID-spectral matched to TAIR10 database

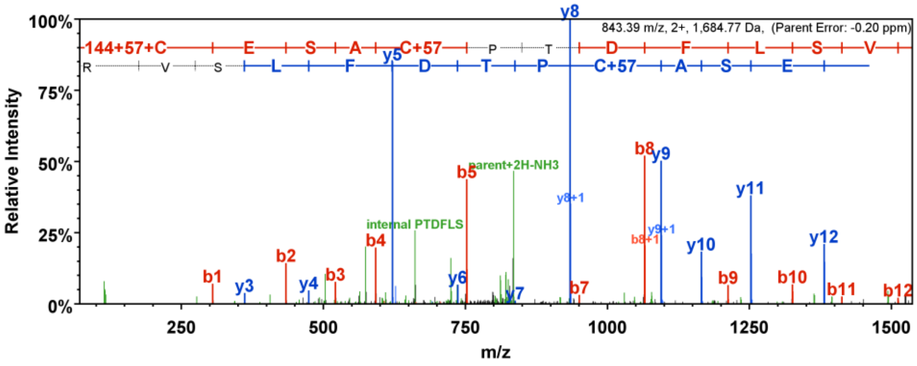

4. ATCG00020.1 [| Symbols: PSBA | photosystem II reaction center protein A

a) Matched peptide: **ETTENESANEGYR**

HCD-spectral matched to TAIR10 database

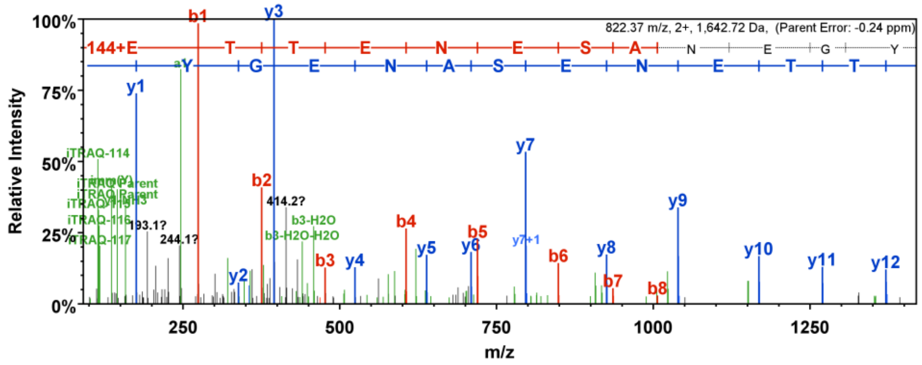

CID-spectral matched to TAIR10 database

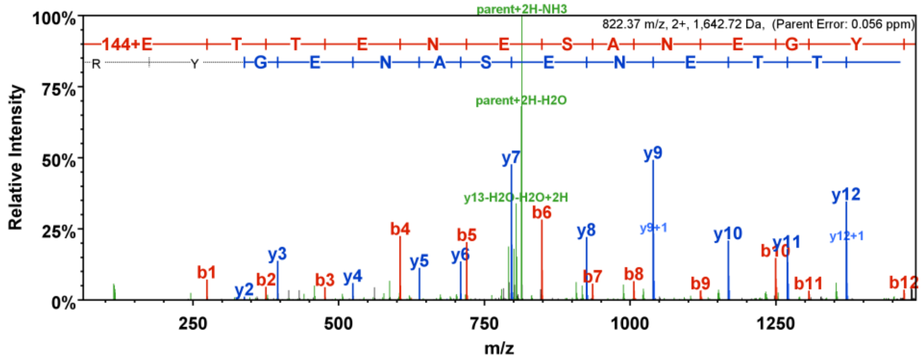

b) Matched peptide: **LIFQYASFNNSR**

HCD-spectral matched to TAIR10 database

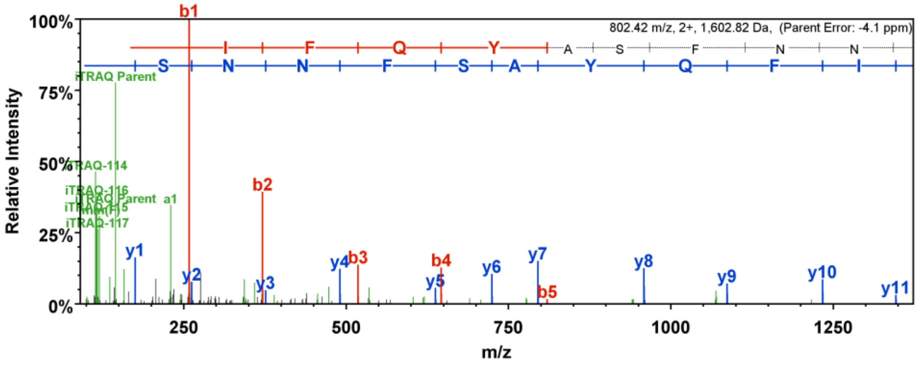

CID-spectral matched to TAIR10 database

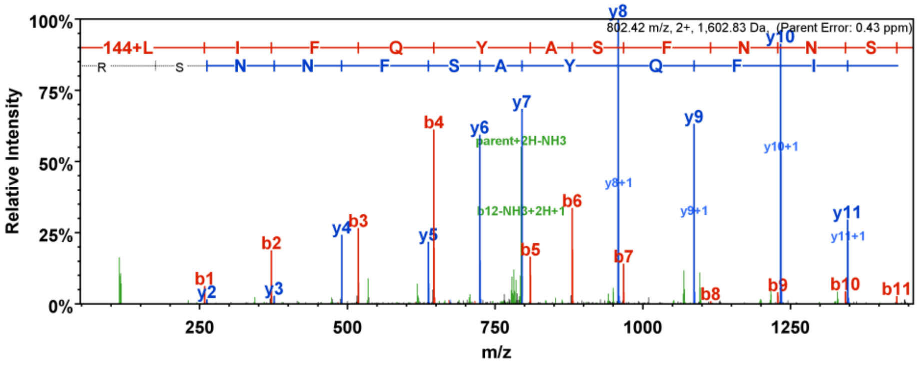

Supplement: Supplementary file 1 — Table S1. Proteases accumulation in seedlings of different genotypes. Table S2. Primers used in the study. Table S3. Peptides identified of CLPC1 in SVR7-GFP CO-IP experiment. Figure S1. Relative expression levels of sense transcripts in the clpc1 mutant and its complementation lines. Figure S2. Over-expressing CLPC2 in clpc1 mutant partially or fully restored the chloroplast RNA level. Figure S3. Schematic diagram to show the plant materials we used. Supplementary dataset 1. Spectral examples of 4 proteins from our iTRAQ-based proteomics analysis. (PDF 2601 kb) [file 12870_2018_1396_MOESM1_ESM.pdf]
